# Dual Empagliflozin and Sacubitril/Valsartan Therapy Improves Ex Vivo Cardiac Function in a Rat Model of Heart Failure

**DOI:** 10.3390/biomedicines14051115

**Published:** 2026-05-14

**Authors:** Maja Murić, Ivan Srejović, Marko Ravić, Jovana Joksimović Jović, Jasmina Sretenović, Marina Nikolić, Nevena Lazarević, Marijana Andjić, Aleksandar Kočović, Sergey Bolevich, Vladimir Jakovljević, Jovana Novaković

**Affiliations:** 1 Department of Physiology, Faculty of Medical Sciences, University of Kragujevac, 34000 Kragujevac, Serbia; majanikolickg90@gmail.com (M.M.); jovana987joksi@gmail.com (J.J.J.); drj.sretenovic@gmail.com (J.S.); marina.nikolic@fmn.kg.ac.rs (M.N.); drvladakgbg@yahoo.com (V.J.); 2 Center of Excellence for the Study of Redox Balance in Cardiovascular and Metabolic Disorders, Faculty of Medical Sciences, University of Kragujevac, 34000 Kragujevac, Serbia; markoravic@hotmail.com (M.R.); nevenasdraginic@gmail.com (N.L.); andjicmarijana10@gmail.com (M.A.); salekkg91@gmail.com (A.K.); jovana.novakovic@fmn.kg.ac.rs (J.N.); 3 Department of Pharmacology, First Moscow State Medical University I.M. Sechenov, 119435 Moscow, Russia; 4 Department of Pharmacy, Faculty of Medical Sciences, University of Kragujevac, 34000 Kragujevac, Serbia; 5 Department of Human Pathology, First Moscow State Medical University I.M. Sechenov, 119435 Moscow, Russia; bolevich2011@yandex.ru

**Keywords:** heart failure, empagliflozin, sacubitril/valsartan, ex vivo cardiac function, gene expression

## Abstract

**Background/Objectives:** This study aimed to clarify the cardioprotective effects of combined empagliflozin and sacubitril/valsartan therapy in an experimental rat model of heart failure (HF). The main research question was whether dual treatment provides greater functional and molecular benefit than either monotherapy, with particular emphasis on oxidative stress, inflammation, apoptosis, and JAK2/STAT3 signaling. **Methods:** HF was induced in rats by 7-day isoproterenol administration and confirmed four weeks later by echocardiographic evidence of reduced ejection fraction (<55%). The animals were then assigned to healthy control, untreated HF, empagliflozin, sacubitril/valsartan, and combined empagliflozin/sacubitril/valsartan groups. Following four weeks of treatment, ex vivo cardiac function was evaluated using the Langendorff technique. Serum cardiospecific markers and natriuretic peptides were measured by ELISA. Oxidative stress parameters were determined in coronary venous effluent, while myocardial gene expression of selected (anti)oxidative, (anti)inflammatory, (anti)apoptotic, and signaling markers was assessed by RT-PCR. Myocardial collagen content was evaluated using Picrosirius red staining. **Results:** HF rats exhibited impaired ex vivo myocardial function, elevated cardiac injury markers, increased oxidative stress, upregulation of pro-inflammatory and pro-apoptotic genes, activation of JAK2/STAT3 signaling, and increased myocardial collagen content. Both monotherapies produced partial benefit. In contrast, combined treatment achieved the most pronounced improvement in contractile performance, attenuated oxidative stress more consistently, reduced expression of TNF-α, IL-1β, IL-6, IL-17, Bax, CASP-3, and CASP-9, favorably modulated JAK2, STAT3, mTOR, and PPARγ expression, and decreased myocardial collagen content. **Conclusions:** Dual empagliflozin and sacubitril/valsartan therapy exerted broader cardioprotective effects than either monotherapy, likely through coordinated antioxidant, anti-inflammatory, anti-apoptotic, and signaling-related mechanisms.

## 1. Introduction

Heart failure (HF) represents one of the major public health challenges in modern society, affecting approximately 1–2% of the general population and more than 10% of individuals over 70 years of age [1]. As the ultimate consequence of diverse cardiovascular pathologies, HF arises from structural and/or functional cardiac impairment, leaving the heart unable to meet the body’s oxygen demands required for physiological metabolic needs [2,3]. However, the precise biological mechanisms underlying cardiac remodeling and HF progression remain incompletely understood.

Oxidative stress results from an imbalance between reactive oxygen species (ROS) generation and the body’s antioxidant defenses, thereby disrupting redox homeostasis [4]. Growing evidence suggests that disturbances in redox balance significantly contribute to cardiac remodeling by inducing hypertrophic, apoptotic and necrotic signaling [5]. Apoptosis, a form of programmed cell death, substantially contributes to chronic cardiomyocyte loss in HF, while ROS activate both the extrinsic death receptor pathway and the intrinsic mitochondrial pathway involved in myocardial cell death [6]. Furthermore, excessive ROS production is closely associated with inflammatory responses within the myocardium. During ischemia–reperfusion injury (IRI), increased ROS levels activate inflammatory cascades and fibroblast progenitors, promoting collagen deposition, fibrosis, and progressive left ventricular (LV) dysfunction [7]. In advanced HF, increased levels of pro-inflammatory cytokines are strongly associated with disease severity.

The molecular pathology of HF is highly complex and involves numerous dysregulated signaling pathways that drive hypertrophy, fibrosis, and cardiomyocyte loss, ultimately impairing cardiac function [8]. Janus kinases (JAKs) are intracellular tyrosine kinases that, together with transcription factors known as signal transducer and activator of transcription (STATs), regulate various aspects of cellular functions [9,10]. JAK/STAT signaling, activated by cytokines, mechanical stress, or myocardial injury, plays a crucial role in cardiomyocyte hypertrophy, cardioprotection, and fibrosis [11,12]. JAK2 phosphorylation has been implicated in several cardiovascular diseases, including myocardial fibrosis and IRI [13,14]. Following phosphorylation, JAK2 recruits and phosphorylates STAT3, whose dimer then translocates to the nucleus and regulates the expression of various downstream genes [15]. The role of JAK2/STAT3 phosphorylation has been investigated in relation to myocardial apoptosis and inflammation; however, its involvement in HF with reduced EF (HFrEF) remains insufficiently explored [16,17].

Relatively novel therapies for HFrEF, including sodium–glucose cotransporter 2 (SGLT2) inhibitors and angiotensin receptor and neprilysin inhibitor (ARNI), have significantly improved clinical management [18,19,20]. Nevertheless, limited data exist regarding the molecular mechanisms underlying their potential synergistic effects when used in combination. In our previous study, we demonstrated enhanced cardioprotective effects of combined empagliflozin and sacubitril/valsartan therapy in rats with isoproterenol (ISO)-induced HF compared with either monotherapy. These beneficial effects were largely driven by potent antioxidant activity, likely resulting from the complementary action of these two agents [21]. In the present study, we aimed to further explore the background of these observations using the ex vivo Langendorff heart technique. Specifically, we focused on ex vivo assessment of cardiac function following chronic treatment with empagliflozin (SGLT2 inhibitor) and sacubitril/valsartan (ARNI), and their combination. Additionally, we examined the dynamics of oxidative stress parameters in coronary venous effluent (CVE), as well as the gene expressions of selected (anti)oxidative, (anti)inflammatory, and (anti)apoptotic markers.

## 2. Materials and Methods

### 2.1. Study Design

This study was conducted at the Center of Excellence for the Study of Redox Balance in Cardiovascular and Metabolic Disorders of the Faculty of Medical Sciences, University of Kragujevac (Serbia). The experimental protocol was approved by the Ethical Committee for Laboratory Animal Welfare of the Faculty of Medical Sciences, University of Kragujevac, Serbia (approval No.: 01-12766, date: 16 November 2023). All procedures adhered to the European Directive for the Welfare of Laboratory Animals (2010/63/EU) and principles of Good Laboratory Practice (86/609/EEC).

**Figure 1:**
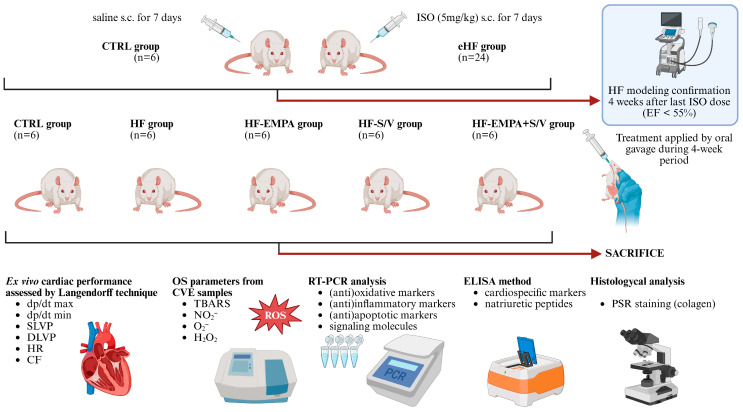
Experimental study design. Abbreviations: ISO, isoproterenol; s.c., subcutaneous; CTRL, control group; eHF, experimental heart failure group; EF, ejection fraction; HF, heart failure group; HF-EMPA, HF group treated with empagliflozin; HF-S/V, HF group treated with sacubitril/valsartan; HF-EMPA+S/V, HF group treated with empagliflozin and sacubitril/valsartan; dp/dt max, maximum rate of LV pressure development; dp/dt min, maximum rate of LV pressure development; SLVP, systolic left ventricular pressure; DLVP, diastolic left ventricular pressure; HR, heart rate; CF, coronary flow; OS, oxidative stress; CVE, coronary venous effluent; TBARS, thiobarbituric acid reactive substances; NO_2_^−^, nitrites; O_2_^−^, superoxide anion radical; H_2_O_2_, hydrogen peroxide; PSR, Picrosirius red; RT-PCR, real-time polymerase chain reaction; ELISA, enzyme-linked immunosorbent assay; ROS, reactive oxygen species.

A total of 30 Wistar Albino male rats, 10–12 weeks of age, with body weight (BW) of 250 ± 20 g, were obtained from the Animal House of the Military Medical Academy (Belgrade, Serbia). Animals were housed in polyethylene cages (six per cage) under standardized, controlled environmental conditions (room temperature 21 ± 2 °C, humidity 55 ± 5%, and 12 h dark–light cycle), with ad libitum access to standard chow and water. The experimental design is presented in Figure 1.

Following a one-week acclimation period, rats were randomly assigned to a healthy control group (CTRL, n = 6) or an ISO-induced HF group (eHF, n = 24). HF was induced in the eHF group by administering ISO (Sigma Aldrich, Taufkirchen, Germany) subcutaneously at a dose of 5 mg/kg of BW dissolved in saline for 7 consecutive days, as we previously described [21]. Four weeks after the final ISO dose, we confirmed HF modeling with transthoracic echocardiography (TTE) showing a significant decline in EF (<55%) in the eHF group compared to the CTRL group, consistent with previous findings [21,22].

The eHF rats were then randomly allocated into four subgroups (n = 6 each), according to the 4-week treatment protocol: HF group (no treatment), HF-EMPA group (empagliflozin 10 mg/kg/day), HF-S/V group (sacubitril/valsartan 68 mg/kg/day), and HF-EMPA+S/V group (empagliflozin 10 mg/kg/day + sacubitril/valsartan 68 mg/kg/day). Empagliflozin (Jardiance^®^, Boehringer Ingelheim, Ingelheim am Rhein, Germany) and sacubitril/valsartan (Entresto^®^, Novartis Pharma AG, Basel, Switzerland) dosing regimens were selected based on previous studies [23,24]. CTRL rats received an equivalent volume of saline subcutaneously to ensure consistent handling. BW was monitored daily, and drug doses were adjusted accordingly.

### 2.2. Ex Vivo Cardiac Function

After the 4-week treatment protocol, all animals were anesthetized with isoflurane inhalation anesthesia and sacrificed by decapitation. An isolated retrograde perfusion of the heart was performed using a Langendorff apparatus (LF-01 F-P, Experimetria Ltd., Budapest, Hungary), as we previously described [25]. After a rapid midline thoracotomy, the hearts were excised. During the procedure, the hearts were continuously irrigated with cold physiological saline (+4 °C). Immediately after removal from the thoracic cavity, each heart was immersed in ice-cold physiological saline (−4 °C to −10 °C) to minimize myocardial metabolic demands (“physiological clamping”), allowing optimal preservation of the myocardium during preparation and cannulation of the ascending aorta. The hearts were carefully transferred and mounted on the Langendorff apparatus via an aortic cannula to enable retrograde perfusion at controlled coronary perfusion pressures (CPPs) ranging from 40 to 120 cmH_2_O. The interval between sacrifice and attaching the heart preparation to the cannula of the Langendorff apparatus did not exceed 30 s for each heart. First, there was a stabilization period of each heart lasting 20 to 25 min at a CPP of 70 cmH_2_O. After stabilization of cardiodynamic parameters and coronary flow (CF) (the same values were measured three times in a row), the experimental protocol of changing CPP was initiated. Krebs–Henseleit solution (NaCl 118 mmol/L, KCl 4.7 mmol/L, CaCl_2_ × 2H_2_O 2.5 mmol/L, MgSO_4_ × 7H_2_O 1.7 mmol/L, NaHCO_3_ 25 mmol/L, KH_2_PO_4_ 1.2 mmol/L, glucose 11 mmol/L, and pyruvate 2 mmol/L) was used as the perfusate, continuously gassed with 95% O_2_ and 5% CO_2_ and maintained at 37 °C (pH 7.4). An incision was made in the left atrium and the mitral valve was disrupted. A pressure transducer (BS473-0184, Experimetria Ltd., Budapest, Hungary) was inserted into the LV to allow continuous monitoring of the following cardiodynamic parameters: maximum rate of LV pressure development (dp/dt max), minimum rate of LV pressure development (dp/dt min), systolic LV pressure (SLVP), diastolic LV pressure (DLVP), and heart rate (HR). CF was measured flowmetrically. During the ex vivo assessment of cardiac function, coronary venous effluent (CVE) was measured at each achieved level of CPP. The CVE samples were stored in appropriate tubes at −20 °C until biochemical analysis.

### 2.3. Determination of Cardiospecific Markers and Natriuretic Peptides via Enzyme-Linked Immunosorbent Assay (ELISA)

Immediately upon sacrifice, blood was collected and the serum was separated by centrifugation. Serum samples were collected and stored at −80 °C for further analysis. In order to investigate myocardial injury in the ISO-induced HF model, we measured the concentrations of cardiospecific markers of myocardial damage from serum samples, including cTnI (pg/mL), creatine kinase MB isoform (CK-MB, pg/mL), and NT-proBNP (pg/mL). Additionally, considering the effects of sacubitril/valsartan on natriuretic peptide levels, concentrations of ANP (pg/mL) and BNP (pg/mL) were also determined. These markers were measured using commercial ELISA kits: (1) Rat TNNI3/cTn-I ELISA Kit (Elabscience, Houston, TX, USA; catalog number: E-EL-R1253), (2) Rat CK-MB ELISA Kit (FineTest, Wuhan, China; catalog number: ER0841), (3) Rat NT-proBNP ELISA Kit (Elabscience, Houston, TX, USA; catalog number: E-EL-R3023), (4) Rat ANP ELISA Kit (FineTest, Wuhan, China; catalog number: ER0738), and (5) Rat BNP ELISA Kit (Elabscience, Houston, TX, USA; catalog number: E-EL-R0126), according to the manufacturer’s instructions.

### 2.4. Determination of Oxidative Stress Markers

In order to evaluate oxidative stress in the LV endocardium and the endothelium of coronary blood vessels, we determined the concentrations of the following pro-oxidative parameters: index of lipid peroxidation (measured as thiobarbituric acid reactive substances—TBARS), NO_2_^−^, O_2_^−^, and H_2_O_2_ in CVE samples, spectrophotometrically (Shimadzu UV 1800 spectrophotometer, Kyoto, Japan). Krebs–Henseleit solution was used as a blank probe.

#### 2.4.1. Determination of Lipid Peroxidation Index (Measured as TBARS)

TBARS was determined by pipetting 200 μL of 0.1 M 1% TBA in NaOH into microtubes, followed by the addition of 800 μL of the sample. The samples were incubated at 100 °C for 15 min, left for 10 min at room temperature, and measured at a wavelength of 530 nm [26].

#### 2.4.2. Determination of NO_2_^−^

NO_2_^−^ was determined by pipetting 1000 μL of the sample, 250 μL of freshly prepared Griess reagent, and 125 μL of NO buffer into a tube. After incubation at room temperature for 15 min, measurement was performed at a wavelength of 550 nm [26].

#### 2.4.3. Determination of O_2_^−^

O_2_^−^ was determined by pipetting 50 μL of the sample into a tube, followed by 950 μL of freshly prepared assay mixture. Measurement was performed with mixing three times at 60 s intervals at a wavelength of 550 nm [26].

#### 2.4.4. Determination of H_2_O_2_

H_2_O_2_ was determined by pipetting 200 μL of the sample into a tube, followed by 800 μL of phenol red solution and 10 μL of peroxidase (POD). After incubation at room temperature for 10 min, measurement was performed at 610 nm [26].

### 2.5. Real-Time Polymerase Chain Reaction (RT-PCR)

Following sacrifice, one-half of the hearts from all animals were collected for RT-PCR analysis. Total RNA was isolated from LV tissue homogenates using TRIzol reagent (Invitrogen, Carlsbad, CA, USA) according to the manufacturer’s instructions. Complementary DNA (cDNA) was synthesized using the High-Capacity cDNA Reverse Transcription Kit (Applied Biosystems, Foster City, CA, USA). Quantitative RT-PCR was performed with Thermo Scientific Luminaris Color HiGreen qPCR Master Mix (Applied Biosystems, Foster City, CA, USA) using mRNA-specific primers for (anti)oxidative markers—endothelial nitric oxide synthase (eNOS) and inducible nitric oxide synthase (iNOS), (anti)inflammatory markers—TNFα, IL-6, IL-1β, IL-17, IL-10, and IL-13, (anti)apoptotic markers—B-cell lymphoma 2 (Bcl-2), Bcl-2-associated X protein (Bax), Caspase-3 (CASP-3), and Caspase-9 (CASP-9), and signaling molecules—Janus kinase 2 (JAK2), signal transducer and activator of transcription (STAT3), mammalian target of rapamycin (mTOR), Peroxisome proliferator-activated receptor γ (PPARγ), and β-actin as the housekeeping gene (Table 1). All PCR reactions were conducted in duplicate, and the mean values were used to calculate relative gene expression using the method described by Livak and Schmittgen [27].

### 2.6. Picrosirus Red Staining

After ex vivo experiments, half of the hearts from all animals were collected for histology analysis. Myocardial tissue was fixed in 4% neutral paraformaldehyde, dehydrated through a graded series of ethanol, cleared in xylene, and embedded in paraffin. Tissue sections (5 μm thick) were prepared and stained with Picrosirius red (PSR) to assess collagen content [21]. Histological images were obtained with a Leica DM2500 light microscope (Wetzlar, Germany), equipped with a Leica Flexacam i5 digital camera (Wetzlar, Germany). Morphometric analysis of collagen content was performed using Image Pro-Plus 7.0 software (Media Cybernetics, Rockville, MD, USA).

### 2.7. Statistical Analysis

All data are expressed as mean ± standard deviation (SD) and analyzed using GraphPad Prism 8 (Windows version; GraphPad Software, La Jolla, CA, USA). Data distribution was evaluated with the Shapiro–Wilk test. Based on whether the data were normally distributed, either parametric tests (one-way ANOVA and independent-samples *t*-test) or nonparametric tests (Kruskal–Wallis H and Mann–Whitney U tests) were applied to assess differences between groups. Statistical significance was considered at *p* < 0.05.

## 3. Results

### 3.1. Assessment of Ex Vivo Cardiac Function

Changes in cardiodynamic parameters and CF were monitored in all experimental groups during the autoregulation protocol at various CPP levels ranging from 40 to 120 cmH_2_O.

Across all CPP levels, dp/dt max was markedly reduced in the HF group compared to the CTRL group. The HF-EMPA group showed a significant decrease only at 120 cmH_2_O, while the HF-S/V group exhibited lower dp/dt max at 100 and 120 cmH_2_O compared to the CTRL group. Dual treatment (HF-EMPA+S/V) significantly improved dp/dt max at CPP levels of 60–120 cmH_2_O compared to HF, whereas S/V monotherapy (HF-S/V) increased dp/dt max only at 60 cmH_2_O. Notably, dp/dt max did not differ significantly between HF-EMPA and HF across any CPP values. Analysis of dp/dt max revealed no significant intra-group changes when the hearts underwent a repeated autoregulation protocol (Figure S1).

At CPP values between 60 and 120 cmH_2_O, dp/dt min was significantly elevated in the HF and HF-EMPA+S/V groups compared to the CTRL group. In relation to the HF group, dp/dt min was reduced in HF-EMPA at 80 cmH_2_O and in HF-S/V at 120 cmH_2_O. Regarding dp/dt min, the only notable intra-group change was detected in the HF-S/V group at a CPP of 80 cmH_2_O, where a significant reduction in this parameter occurred during the repeated autoregulation cycle (Figure S2D).

SLVP was significantly decreased in the HF group at 100 and 120 cmH_2_O, whereas no meaningful differences were detected among the other treatment groups. DLVP values did not differ significantly across groups. Analysis of SLVP and DLVP revealed no significant intra-group changes within any group during repeated exposure to the autoregulation protocol (Figures S3 and S4, respectively).

**Figure 2:**
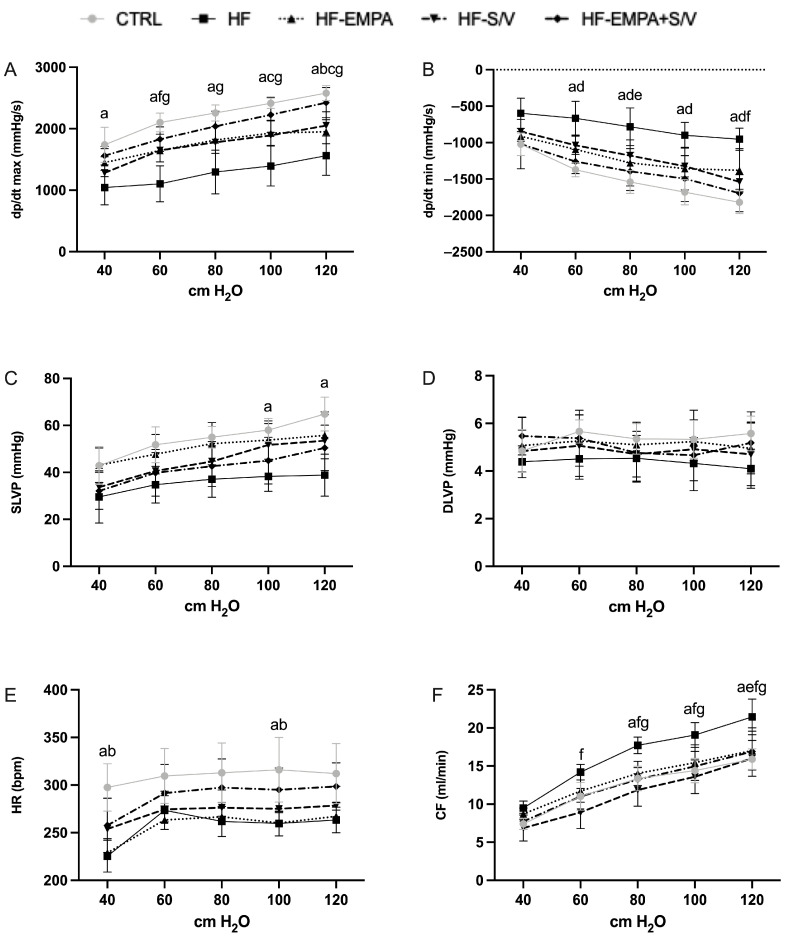
The effects of dual treatment on ex vivo cardiac function of HF rats. (**A**) Maximum rate of left ventricular pressure development (dp/dt max); (**B**) minimum rate of left ventricular pressure development (dp/dt min); (**C**) systolic left ventricular pressure (SLVP); (**D**) diastolic left ventricular pressure (DLVP); (**E**) heart rate (HR); and (**F**) coronary flow (CF). Values are expressed as mean ± SD (n = 6 per group). Statistical significance was expressed as *p* < 0.05. ^a^ CTRL vs. HF; ^b^ CTRL vs. HF-EMPA; ^c^ CTRL vs. HF-S/V; ^d^ CTRL vs. HF-EMPA+S/V; ^e^ HF vs. HF-EMPA; ^f^ HF vs. HF-S/V; ^g^ HF vs. HF-EMPA+S/V.

HR was significantly reduced in both the HF and HF-EMPA groups at CPP levels of 40 and 100 cmH_2_O compared to the CTRL group, with no additional intergroup differences (Figure 2E). During repeated autoregulation, HR was significantly reduced in the HF group at CPP levels of 60 and 80 cmH_2_O (Figure S5). Similarly, the HF-EMPA and HF-EMPA+S/V groups exhibited a significant decrease in HR at a CPP of 60 cmH_2_O (Figure S5E).

CF was markedly increased in the HF group at CPP values of 80–120 cmH_2_O compared to the CTRL group (Figure 2F). In relation to the HF group, CF was significantly lower in HF-S/V at 60–120 cmH_2_O and in HF-EMPA+S/V at 80–120 cmH_2_O, while in the HF-EMPA group, a reduction in CF was observed only at 120 cmH_2_O. During repeated autoregulation, CF was significantly reduced in the HF group at a CPP of 120 cmH_2_O (Figure S6B). In contrast, CF increased significantly in the HF-EMPA group at 120 cmH_2_O and in the HF-S/V group at 100 cmH_2_O (Figure S6C,D, respectively).

### 3.2. Assessment of Cardiospecific Marker Levels

**Figure 3:**
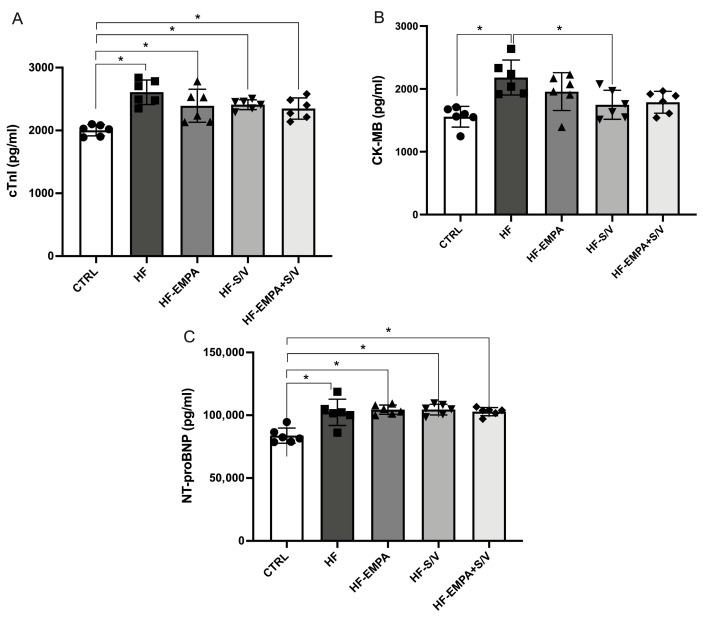
The effects of dual treatment on the concentration of cardiospecific markers in the serum of HF rats. (**A**) Cardiac troponin I (cTnI); (**B**) creatine kinase isoform MB (CK-MB); and (**C**) N-terminal pro B-type natriuretic peptide (NT-proBNP). Values are expressed as mean ± SD (n = 6 per group). * *p* < 0.05.

Following the completion of the experimental protocol, serum concentrations of cardiospecific markers (cTnI, CK-MB, and NT-proBNP) and natriuretic peptides (ANP and BNP) were measured in all groups. The HF group exhibited significantly elevated levels of all cardiac markers compared to the CTRL group (Figure 3A–C). While all therapeutic interventions (HF-EMPA, HF-S/V, and HF-EMPA+S/V) resulted in higher cTnI and NT-proBNP levels compared to the CTRL group, these values did not differ significantly from those in the HF group (Figure 3A,C). Notably, CK-MB levels were significantly decreased only in the HF-S/V group compared to the HF group (Figure 3B).

### 3.3. Assessment of Natriuretic Peptide Levels

**Figure 4:**
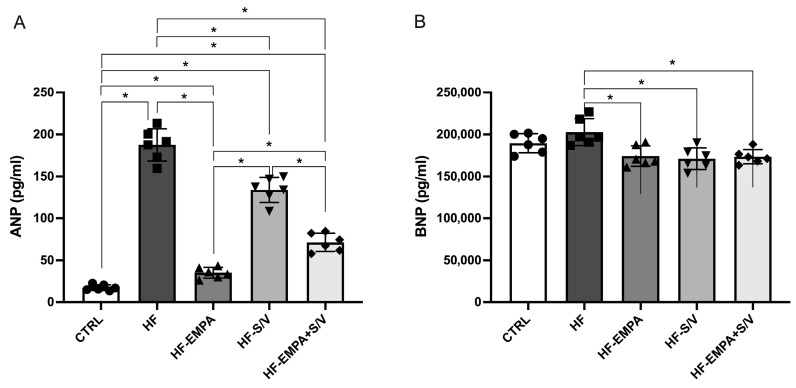
The effects of dual treatment on the concentration of natriuretic peptides in the serum of HF rats. (**A**) Atrial natriuretic peptide (ANP); (**B**) brain-type natriuretic peptide (BNP). Values are expressed as mean ± SD (n = 6 per group). * *p* < 0.05.

ANP concentrations were markedly elevated in the HF group and in all treated groups (HF-EMPA, HF-S/V, and HF-EMPA+S/V) compared to CTRL (Figure 4A). Application of therapeutic protocols significantly lowered ANP levels relative to HF. Among the treated groups, ANP levels were highest in HF-S/V compared to HF-EMPA and HF-EMPA+S/V, and higher in HF-EMPA+S/V than in HF-EMPA. While BNP levels did not differ significantly between CTRL and HF, all therapeutic interventions resulted in a significant reduction in BNP compared to HF (Figure 4B).

### 3.4. Assessment of Oxidative Stress Marker Levels in Coronary Venous Effluent

**Figure 5:**
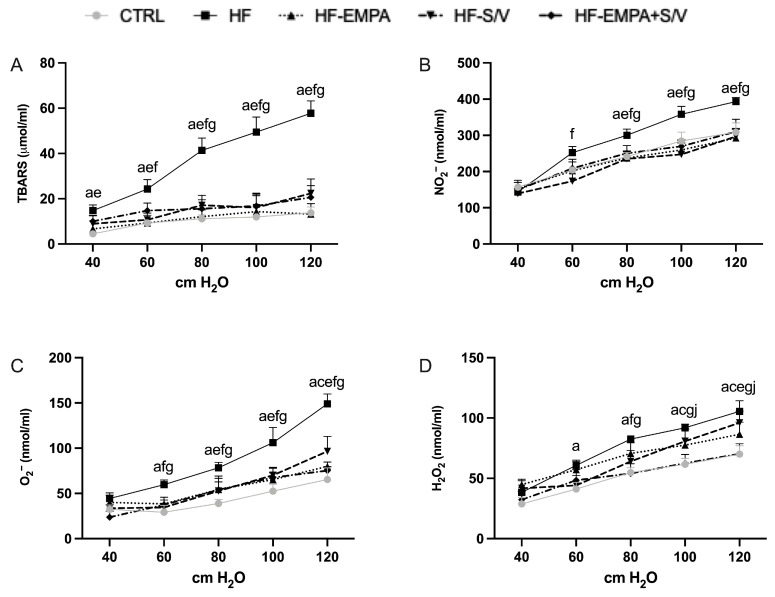
The effects of dual treatment on prooxidative parameters from the CVE of HF rats. (**A**) Index of lipid peroxidation (measured as TBARS); (**B**) nitrites (NO_2_^−^); (**C**) superoxide anion radical (O_2_^−^); and (**D**) hydrogen peroxide (H_2_O_2_). Values are expressed as mean ± SD (n = 6 per group). Statistical significance was expressed as *p* < 0,05. ^a^ CTRL vs. HF; ^c^ CTRL vs. HF-S/V; ^e^ HF vs. HF-EMPA; ^f^ HF vs. HF-S/V; ^g^ HF vs. HF-EMPA+S/V; ^j^ HF-S/V vs. HF-EMPA+S/V.

At the end of the experimental protocol, TBARS levels were markedly elevated in the HF group across all pressure values (40–120 cmH_2_O) compared to CTRL (Figure 5A). TBARS concentrations were significantly decreased by HF-EMPA at all pressures, by HF-S/V at 60–120 cmH_2_O, and by HF-EMPA+S/V at 80–120 cmH_2_O. Intra-group differences during re-exposure of isolated hearts to the autoregulation protocol revealed a significant reduction in TBARS levels in the HF group at 100 and 120 cmH_2_O (Figure S7B). Conversely, TBARS increased in the HF-S/V group (Figure S7D), whereas a decrease was observed in the HF-EMPA+S/V group at 60 cmH_2_O (Figure S7E). No significant changes were detected between consecutive autoregulation protocols in the remaining experimental groups.

NO_2_^−^ levels were significantly higher in HF at 80–120 cmH_2_O versus CTRL (Figure 5B), and all treatment groups effectively reduced NO_2_^−^ at these pressures. Additionally, HF-S/V lowered NO_2_^−^ at 60 cmH_2_O. Re-exposure to the autoregulation protocol led to a significant increase in NO_2_^−^ levels in the HF-EMPA group at 100 and 120 cmH_2_O (Figure S8C). A similar increase was observed in the HF-S/V group (Figure S8D), whereas NO_2_^−^ decreased in the HF-EMPA+S/V group at 100 cmH_2_O (Figure S8E). No significant changes were detected between consecutive autoregulation protocols in the remaining experimental groups.

O^2−^ concentrations were significantly increased in HF at 60–120 cmH_2_O (Figure 5C), with HF-S/V also elevated at 120 cmH_2_O. All treatments decreased O_2_^−^ at 80–120 cmH_2_O, while HF-S/V and HF-EMPA+S/V also reduced it at 60 cmH_2_O. Re-exposure to the autoregulation protocol resulted in significantly higher O_2_^−^ levels in the CTRL group at 60 and 80 cmH_2_O (Figure S9A). Similarly, O_2_^−^ concentrations increased in the HF-EMPA group at 60 cmH_2_O (Figure S9C) and in the HF-S/V group at 60 and 80 cmH_2_O (Figure S9D). No significant changes were observed between consecutive autoregulation protocols in the remaining experimental groups.

H_2_O_2_ was elevated in HF at 60–120 cmH_2_O (Figure 5D), and in HF-S/V at 100–120 cmH_2_O compared to CTRL. HF-EMPA+S/V significantly lowered H_2_O_2_ at 80–120 cmH_2_O, whereas HF-EMPA showed a reduction only at 120 cmH_2_O and HF-S/V at 80 cmH_2_O. H_2_O_2_ was further reduced in HF-EMPA+S/V versus HF-S/V at 100–120 cmH_2_O. Re-exposure to the autoregulation protocol led to a significant decrease in H_2_O_2_ levels in the CTRL group at 80 cmH_2_O (Figure S10A). Conversely, H_2_O_2_ concentrations increased in the HF-S/V group at 60 cmH_2_O (Figure S10D) and in the HF-EMPA+S/V group at 80 and 100 cmH_2_O (Figure S10E) during the repeated protocol. No significant changes were detected between consecutive autoregulation protocols in the remaining experimental groups.

### 3.5. Assessment of Relative Gene Expression of (Anti)Oxidative Markers

**Figure 6:**
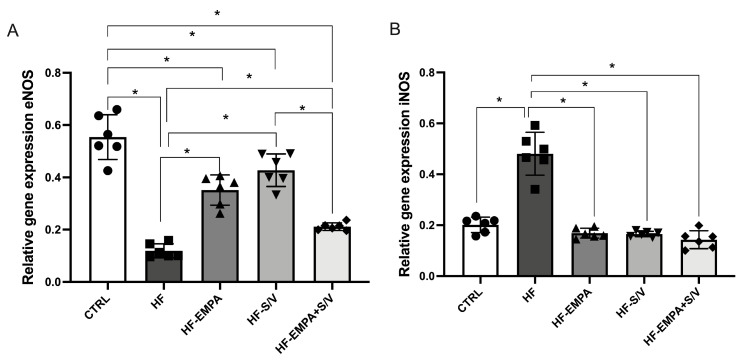
The effects of dual treatment on relative gene expression of (anti)oxidative markers in myocardial tissue of HF rats. (**A**) Relative gene expression of endothelial nitrite oxide synthase (eNOS); (**B**) relative gene expression of inducible nitrite oxide synthase (iNOS). Values are expressed as mean ± SD (n = 6 per group). * *p* < 0.05.

eNOS gene expression was markedly reduced in HF compared to the CTRL group (Figure 6A). All treatment protocols (HF-EMPA, HF-S/V, and HF-EMPA+S/V group) significantly elevated eNOS expression relative to HF, although all remained lower than the CTRL levels. Notably, the HF-EMPA+S/V group displayed the lowest eNOS expression among the treatment groups, reaching statistical significance compared to HF-S/V. Conversely, iNOS expression was significantly upregulated in HF compared to the CTRL group, while all therapeutic interventions effectively decreased iNOS expression relative to the HF group (Figure 6B).

### 3.6. Assessment of Relative Gene Expression of (Anti)Inflammatory Markers

**Figure 7:**
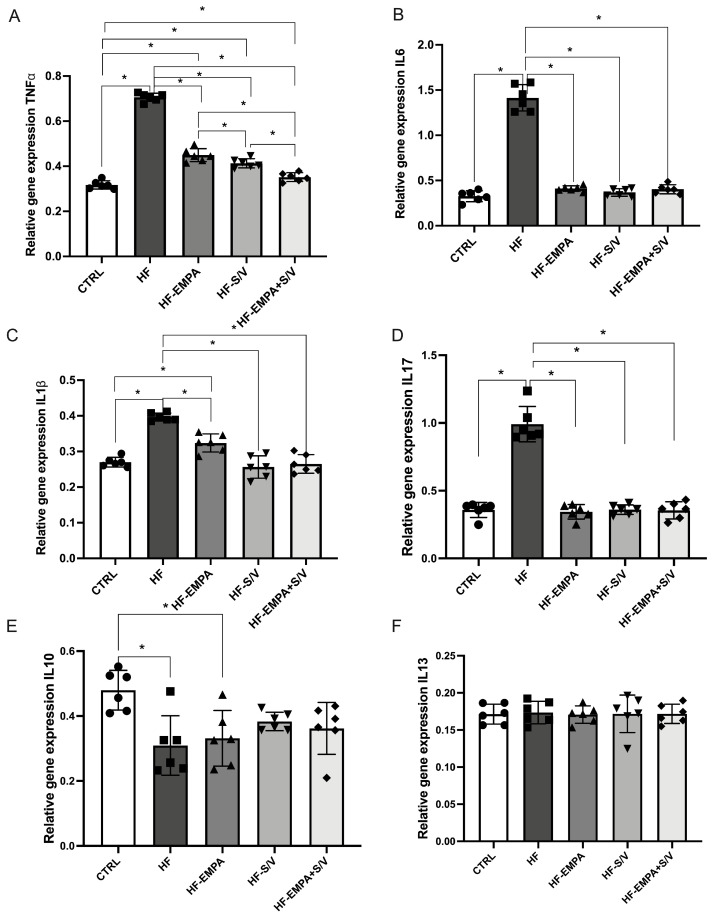
The effects of dual treatment on relative gene expression of (anti)inflammatory markers in myocardial tissue of HF rats. (**A**) Relative gene expression of tumor necrosis factor α (TNFα); (**B**) relative gene expression of interleukin-6 (IL-6); (**C**) relative gene expression of interleukin-1 beta (IL-1β); (**D**) relative gene expression of interleukin-17 (IL-17); (**E**) relative gene expression of interleukin-10 (IL-10); and (**F**) relative gene expression of interleukin-13 (IL-13); Values are expressed as mean ± SD (n = 6 per group). * *p* < 0.05.

In the HF group, the expression of pro-inflammatory genes TNF-α, IL-6, IL-1β, and IL-17 was significantly elevated compared to the CTRL group (Figure 7A–D). All therapeutic interventions (HF-EMPA, HF-S/V, and HF-EMPA+S/V) significantly decreased TNF-α expression relative to the HF group, though the levels remained higher than in the CTRL group. Among the therapies, HF-EMPA exhibited the highest TNF-α expression, whereas combination therapy (HF-EMPA+S/V) was the most effective in reducing TNF-α levels. IL-6 expression was significantly attenuated in all treatment groups compared to the HF group. Similarly, IL-1β expression decreased in all therapeutic groups relative to the HF group, yet HF-EMPA still showed significantly higher levels than the CTRL group, unlike the other treatment groups. IL-17 expression was reduced across all treated groups compared to the HF group. No significant differences in IL-10 expression were noted between HF and the treated groups, though HF-EMPA exhibited lower IL-10 levels than the CTRL group (Figure 7E). IL-13 expression remained unchanged across all experimental groups (Figure 7F).

### 3.7. Assessment of Relative Gene Expression of (Anti)Apoptotic Markers

**Figure 8:**
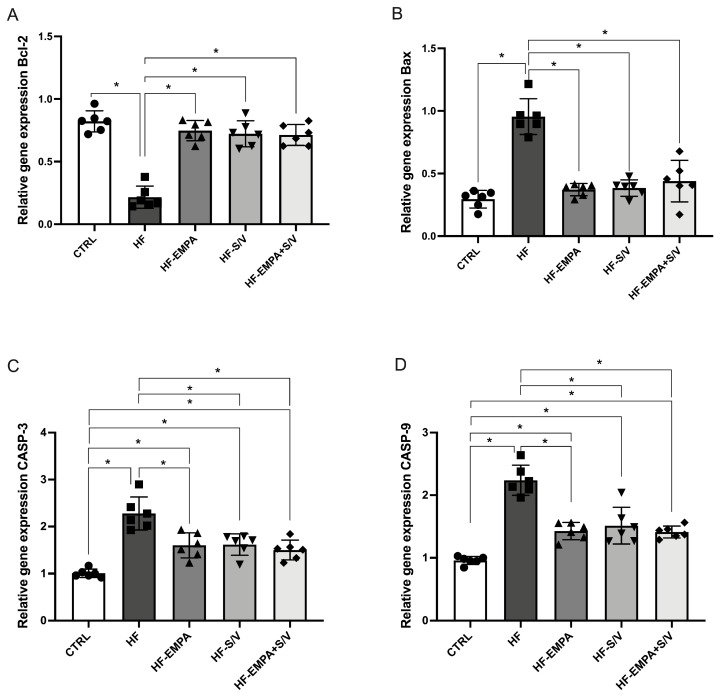
The effects of dual treatment on relative gene expression of (anti)apoptotic markers in myocardial tissue of HF rats. (**A**) Relative gene expression of B-cell lymphoma 2 (Bcl-2); (**B**) relative gene expression of Bcl-2-associated X protein (Bax); (**C**) relative gene expression of Caspase-3 (CASP-3); and (**D**) relative gene expression of Caspase-9 (CASP-9). Values are expressed as mean ± SD (n = 6 per group). * *p* < 0.05.

In this study, we evaluated the relative gene expression of pro- and anti-apoptotic markers in rats with HF and assessed the effects of the applied therapeutic protocols. The HF group exhibited reduced Bcl-2 expression compared to the CTRL group (Figure 8A). All therapeutic interventions (HF-EMPA, HF-S/V, and HF-EMPA+S/V) significantly increased Bcl-2 expression relative to the HF group. Conversely, Bax expression was significantly elevated in HF compared to the CTRL group, while all three treatments effectively reduced Bax expression (Figure 8B).

The relative expression of CASP-3 and CASP-9 was also significantly higher in HF compared to the CTRL group (Figure 8C,D). Although all therapeutic protocols maintained CASP-3 and CASP-9 expression levels above CTRL, they significantly attenuated these markers compared to the HF group.

### 3.8. Assessment of Relative Gene Expression of Specific Signaling Molecules

**Figure 9:**
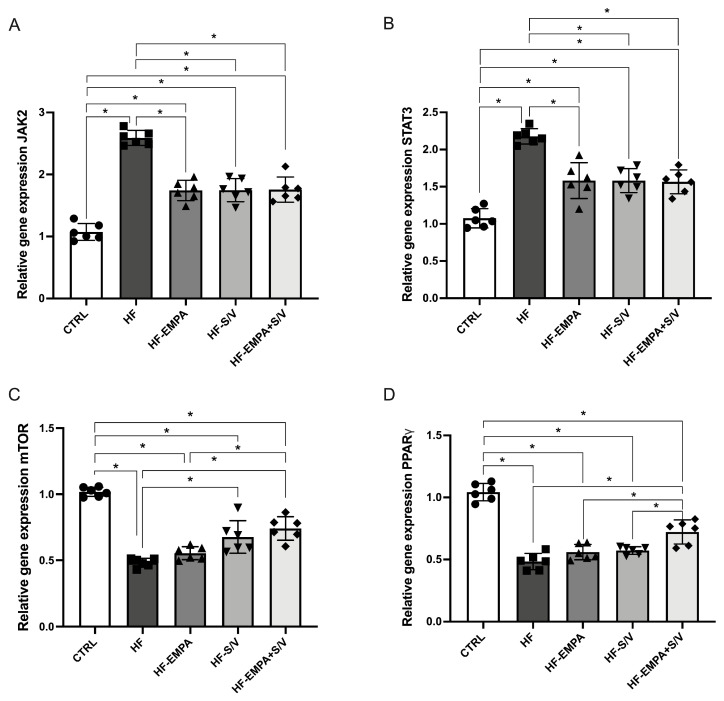
The effects of dual treatment on the relative gene expression of specific signaling molecules in the myocardial tissue of HF rats. (**A**) Relative gene expression of Janus kinase 2 (JAK2); (**B**) relative gene expression of signal transducer and activator of transcription 3 (STAT3); (**C**) relative gene expression of mammalian target of rapamycin (mTOR); and (**D**) relative gene expression of peroxisome proliferator-activated receptor gamma (PPARγ). Values are expressed as mean ± SD (n = 6 per group). * *p* < 0.05.

In this study, the relative gene expression of specific signaling molecules was evaluated in rats with heart failure, along with the effects of different therapeutic protocols. The results showed that JAK2 expression was significantly elevated in the HF group compared to CTRL (Figure 9A). All therapeutic protocols (HF-EMPA, HF-S/V, and HF-EMPA+S/V) significantly reduced JAK2 expression relative to HF, although these values remained higher than in CTRL (Figure 9A).

Similarly, STAT3 expression was significantly increased in the HF group and in all treated groups compared to CTRL (Figure 9B). The applied therapeutic interventions effectively decreased STAT3 expression relative to HF.

Conversely, mTOR expression was significantly reduced in the HF group and in all therapy-treated groups compared to CTRL (Figure 9C). However, mTOR expression was significantly higher in the HF-S/V and HF-EMPA+S/V groups compared to HF, whereas no such increase was observed in the HF-EMPA group. Moreover, the HF-EMPA group exhibited significantly lower mTOR expression compared to HF-EMPA+S/V.

PPARγ expression was significantly decreased in the HF group and in all treated groups compared to CTRL (Figure 9D). Interestingly, PPARγ expression was increased only in the HF-EMPA+S/V group relative to HF, while HF-EMPA and HF-S/V groups showed significantly lower expression compared to HF-EMPA+S/V.

### 3.9. Assessment of Collagen Content in Heart Tissue

**Figure 10:**
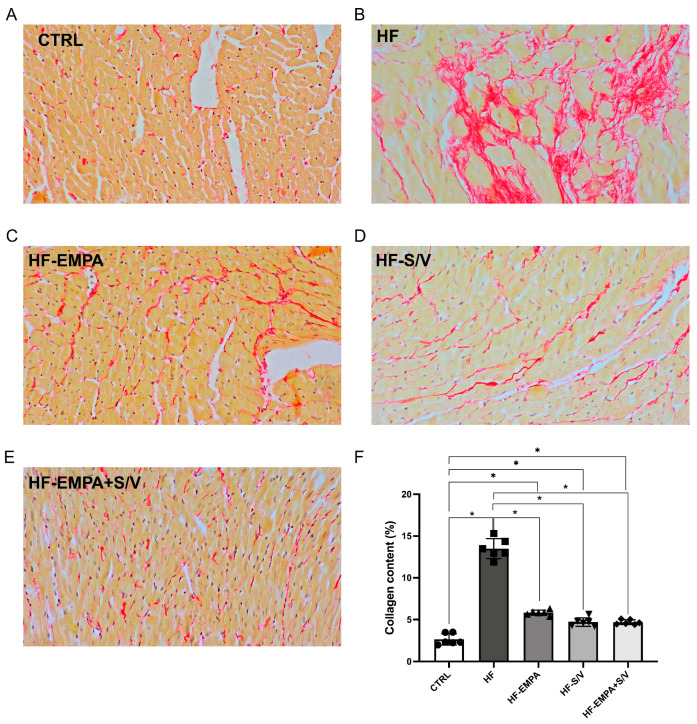
The effects of dual treatment on collagen content in the heart. (**A**–**E**) Representative Picrosirius red (PSR)-stained heart tissue sections (200× magnification). (**F**) Myocardial collagen content. Abbreviations: CTRL, control group; HF, heart failure (HF) group without treatment; HF-EMPA, HF group treated with empagliflozin; HF-S/V, HF group treated with sacubitril/valsartan; HF-EMPA+S/V, HF group treated with empagliflozin and sacubitril/valsartan. Values are expressed as mean ± SD (n = 6 per group). * *p* < 0.05.

During PSR staining, collagen fibers appeared red against the yellow-stained myocardial tissue (Figure 10A–E). Cardiac collagen content was significantly higher in the HF group (an increase of 481%) in comparison to the CTRL group (Figure 10F). All three therapeutic protocols led to a significant decrease in collagen content (2–3-fold) in comparison to the HF group. However, myocardial collagen content in the treated groups was still significantly increased in comparison to the CTRL group.

## 4. Discussion

HF remains one of the leading causes of hospitalization and mortality worldwide, affecting more than 64 million people globally [28]. In previous years, results from large clinical trials have substantially improved therapeutic guidelines for HF management, leading to the incorporation of new drug classes, SGLT2 inhibitors and ARNI, into the standard HFrEF treatment [18]. Nevertheless, the effects of concomitant administration of both drug classes remain under extensive investigation. Thus, the aim of this study was to dissect in more detail the effects of SGLT2 inhibitors and ARNI as monotherapies, as well as their combination, in an ISO-induced experimental model of HFrEF.

In the present ex vivo Langendorff model, ISO-induced HF resulted in marked impairment of myocardial function under controlled perfusion conditions. Both empagliflozin and sacubitril/valsartan monotherapies produced partial improvements in cardiac performance, whereas the combined treatment consistently showed the most pronounced functional recovery across multiple CPPs (Figure 2). Untreated HF rats developed significant deterioration of ex vivo myocardial function. This was evidenced by decreased dp/dt max at all CPPs, SLVP at CPPs of 100 and 120 cmH_2_O, and HR at CPPs of 40 and 100 cmH_2_O, along with elevated dp/dt min at CPPs of 60–120 cmH_2_O and CF at CPPs of 80–120 cmH_2_O. Both empagliflozin and sacubitril/valsartan monotherapy had modest effects on cardiac performance; however, the dual treatment produced the most prominent cardioprotective effects, significantly enhancing dp/dt max across a wider CPP range. Previous research has demonstrated reduced SERCA2a expression in HF, leading to impaired Ca^2+^ handling and weakened myocardial contractility [29]. Low-dose empagliflozin induced an increase in dp/dt max and a decrease in dp/dt min in healthy rats compared with pre-treatment values [30]. These effects of empagliflozin could be partially explained by the upregulation of SERCA2a and the downregulation of MMP9 and the sodium–hydrogen exchanger 1 (NHE1) [30]. Assessment of the effects of sacubitril/valsartan in mice with obesity-associated cardiomyopathy using the Langendorff technique showed significant improvement in heart function, reflected by a reduction in end-diastolic pressure, improved heart response to workload (induced by increased CaCl_2_ concentration in perfusion solution) and enhanced cardiac energetics [31]. Also, in our previous work, the cardioprotective effects of sacubitril/valsartan were shown in hypertension-induced hypertrophic cardiomyopathy [32]. The results indicated that all monitored cardiodynamic parameters, examined using a similar experimental protocol with stepwise changes in CPPs in Langendorff retrogradely perfused hearts, in the sacubitril/valsartan-treated group were significantly closer to those of healthy animals. In the present study, we demonstrated that combined administration of empagliflozin and sacubitril/valsartan exhibited an even more pronounced protective effect, suggesting additive cardioprotective potential (Figure 2).

In our study, biochemical evidence of myocardial damage supported the functional findings. Elevations in cTnI, CK-MB, and NT-proBNP levels in the HF group confirm substantial structural injury and neurohormonal activation (Figure 3). Although none of the therapeutic protocols normalized all biomarkers, HF-S/V significantly reduced CK-MB levels, while all treatments lowered ANP and BNP in comparison to the HF group (Figure 4). Such findings may suggest that the therapy applied in our study, although exerting beneficial effects on ex vivo myocardial function, may not be sufficient to fully reverse the biochemical imbalance in HF rats. Interestingly, contrary to ANP, BNP levels were unchanged between the CTRL and HF groups, but decreased with all treatments, indicating different responsiveness of natriuretic peptides to pharmacological intervention in this model. A previous study showed dose-dependent protective effects of empagliflozin on myocardial fibrosis and cardiospecific markers, including cTnI and CK-MB, in ISO-induced HF [33]. In this study, increases in cTnI and CK-MB were more pronounced compared to our study, probably due to longer exposition to ISO; the less pronounced decrease in cardiospecific markers in our study may be a consequence of the administration of the drugs only after confirmed HF, while in the aforementioned study, empagliflozin was applied simultaneously with ISO [33].

Oxidative stress analyses revealed profound pro-oxidative imbalance in HF rats, characterized by elevated TBARS, NO_2_^−^, O_2_^−^, and H_2_O_2_ concentrations across nearly all CPP values (Figure 5). These findings align with the known role of ROS overproduction in ISO-induced cardiotoxicity [34]. The most effective reduction in TBARS levels was observed in the HF-EMPA group at all CPP values, whereas sacubitril/valsartan-treated groups (HF-S/V and HF-EMPA+S/V) were effective in lowering TBARS primarily at higher CPP values compared to the HF group. All therapeutic protocols contributed to a decrease in NO_2_^−^ and O_2_^−^ concentrations at higher CPPs, with sacubitril/valsartan monotherapy (HF-S/V group) being the most effective in reducing NO_2_^−^, and both sacubitril/valsartan-treated groups (HF-S/V and HF-EMPA+S/V) showing the most pronounced reduction in O_2_^−^ compared to the HF group. Dual therapy (HF-EMPA+S/V group) demonstrated the greatest efficacy in reducing H_2_O_2_, especially at high CPP values (Figure 5). Improvement of oxidative stress by empagliflozin and sacubitril/valsartan may be partially related to improvement of cardiac energetics and mitochondrial function, and consequent reduction of ROS production [31,33]. Also, empagliflozin prevents NOX activation and ROS production by inhibiting protein kinase C activity in human coronary artery endothelial cells exposed to mechanical stress, an effect mediated by inhibition of the NHE/Na^+^/Na^+^/Ca^2+^ exchanger/Ca^2+^ cascade [35]. An important mediating mechanism in the improvement of redox balance by empagliflozin and sacubitril/valsartan is increased capacity of the antioxidative defense system, as shown by us and others [23,36,37]. In a rat model of pressure-overload-induced HF, sacubitril/valsartan was shown to alleviate oxidative stress and apoptosis via regulation of the Sirtuin 3/MnSOD signaling cascade [38].

Inflammation is closely related to oxidative stress and dynamic of ROS production. ISO induced a significant increase in iNOS expression and a simultaneous decrease in eNOS expression (Figure 6). All therapeutic options improved the iNOS/eNOS expression ratio, but sacubitril caused the greatest increase in eNOS expression, while the drug combination caused the greatest decrease in eNOS expression (Figure 6). In a study conducted by Deger and colleagues, ISO administration in rats increased both iNOS and eNOS expression, accompanied by cardiomyocyte damage and myocardial necrosis [39]. This suggests that the antioxidative effects of the combined treatment may not rely primarily on eNOS upregulation but rather involve additional mechanisms such as reduced oxidative stress and improved cellular redox handling. Consistent with previous reports linking iNOS activation to inflammatory signaling and adverse HF outcomes [40], all treatments significantly reduced iNOS expression compared to HF. Although no significant differences were observed between the treated groups, dual therapy showed a tendency toward the greatest reduction in iNOS expression. It has been shown that inflammation plays a central role in the progression of cardiorenal syndrome, as evidenced by marked upregulation of pro-inflammatory mediators in rats with cardiorenal syndrome. Therapeutic intervention with empagliflozin and sacubitril/valsartan significantly attenuated the inflammatory response, while combined therapy exerted the most pronounced anti-inflammatory effects compared with either monotherapy [41].

In HFrEF, inflammation develops as a consequence of an “index” event and peripheral tissue hypoperfusion [42]. In our study, ISO administration caused a significant increase in pro-inflammatory cytokines (TNF-α, IL-6, IL-1β, and IL-17) in the HF group compared to the CTRL group (Figure 7). Although in all three therapeutic protocols (HF-EMPA, HF-S/V, and HF-EMPA+S/V groups), the level of TNF-α gene expression remained increased compared to the CTRL group, the expression of this gene was significantly lower compared to the HF group, with the greatest efficacy observed during combined therapy (HF-EMPA+S/V group). Furthermore, all therapeutic protocols in our study (HF-EMPA, HF-S/V, and HF-EMPA+S/V groups) led to significantly lower gene expression of IL-6, IL-1β, and IL-17 compared to the HF group. Regarding the expression of anti-inflammatory cytokine genes (IL-10 and IL-13), a significantly lower expression of IL-10 was observed in the HF group compared to the CTRL group, whereas the applied therapeutic protocols did not show a significant effect on anti-inflammatory cytokines compared to the HF group (Figure 7). This may indicate that the anti-inflammatory effects of applied treatments are mediated primarily through suppression of pro-inflammatory mediators rather than activation of anti-inflammatory cytokines.

The HF group had decreased gene expression of Bcl-2, with increased Bax, CASP-3, and CASP-9. All three therapeutic strategies significantly reversed these changes (Figure 8). The results of previous studies have shown that the administration of empagliflozin prevents cell death in models of I/R injury and diabetic cardiomyopathy by activating anti-apoptotic factors and reducing ROS-dependent cell death, as well as by modulating the ratio of pro- and anti-apoptotic molecules [43]. In addition, several preclinical studies have demonstrated that sacubitril/valsartan (S/V) exerts anti-apoptotic effects manifested by reduced expression of apoptotic markers such as Bax and Bcl-2, as well as decreased caspase-3 activity, indicating its potential for myocardial protection. Although we observed differences among the treated groups in relation to the gene expression of the examined (anti)apoptotic markers, the dual therapy tended to be the most effective in reducing the gene expression of Bax, CASP-3, and CASP-9, suggesting a potential capacity of this therapeutic approach to modulate apoptotic pathways and protect against cell death in heart failure.

JAK2 and STAT3 expressions were significantly elevated in HF rats (Figure 9), aligning with their known activation in dilatative cardiomyopathy [44]. Previous studies have indicated potential effects of empagliflozin on the JAK2/STAT3 signaling cascade. Specifically, empagliflozin treatment significantly reduced IL-6 levels in patients with type 2 cardiorenal syndrome, which was accompanied by an increase in JAK2 and STAT3 levels [45]. These results suggest that empagliflozin may exert anti-inflammatory effects associated with the activation of JAK2/STAT3 signaling pathways [45]. Furthermore, in an experimental H/R model of isolated cardiomyocytes exposed to high-glucose conditions, empagliflozin activated the JAK2/STAT3 signaling pathway, contributing to the protection of cardiomyocytes from H/R injury [46]. However, after STAT3 expression was reduced, the protective effect of empagliflozin was attenuated, highlighting the importance of this pathway in its cardioprotective activity [46]. On the other hand, the direct effects of sacubitril/valsartan on JAK2/STAT3 signaling have not been sufficiently investigated. While all treatments suppressed JAK2 and STAT3 upregulation, the most notable finding was the simultaneous modulation of mTOR and PPARγ, suggesting a cross-talk between metabolic and inflammatory signaling pathways. The HF group had decreased gene expression of both markers, while sacubitril/valsartan (HF-S/V and HF-S/V+EMPA)—but not empagliflozin alone (HF-EMPA)—elevated mTOR expression, and dual therapy further amplified this effect. Since mTOR signaling enhances cellular survival and protein synthesis, its upregulation likely contributes to improved functional recovery observed ex vivo [47]. Conversely, PPARγ expression was restored only by the dual treatment (HF-EMPA+S/V), indicating improved metabolic flexibility and anti-inflammatory transcriptional control. Taken together, these findings suggest that the synergistic cardioprotective effect of dual therapy arises from a coordinated downregulation of JAK2/STAT3 inflammatory signaling and simultaneous enhancement of mTOR and PPARγ pathways.

The obtained results indicate that ISO-induced HF leads to a marked increase in myocardial collagen deposition, as evidenced by a 481% elevation in the HF group compared to the control group (Figure 10). This finding is consistent with well-established mechanisms of cardiac remodeling, characterized by fibrosis and structural deterioration of myocardial tissue [21,48]. Treatment with empagliflozin, sacubitril/valsartan, and their combination significantly reduced collagen content (by 2–3-fold) compared to the untreated HF group, suggesting a pronounced anti-fibrotic effect of these therapeutic strategies. These results are in accordance with previous findings that showed anti-fibrotic effects of empagliflozin [49], as well as sacubitril/valsartan [50], on cardiac tissue. However, despite this improvement, collagen fiber content in all treated groups remained significantly higher than in the control group, indicating that fibrosis was only partially reversed in our study. Such a result may imply that a longer treatment duration or earlier therapeutic intervention is likely required to achieve a more complete restoration of myocardial structure. Notably, while all treatments were effective, the potentially superior effect of combined therapy was observed.

The main clinical significance of the obtained results is reflected in the confirmation of a more pronounced protective effect of the combined use of empagliflozin and sacubitril/valsartan, as well as in a better understanding of the beneficial effects of their combined use. The key shortcoming of this research is the lack of a deeper confirmation of the results through the deeper examination of the interrelation of oxidative stress, inflammation and signaling molecules.

## 5. Conclusions

Our study provides an additional mechanistic insight into the cardioprotective effects of dual empagliflozin and sacubitril/valsartan treatment in an ISO-induced HF model. Building upon our previous findings of cardioprotection due to enhanced antioxidative synergy between these agents, we extend this evidence by demonstrating that this dual treatment not only improves ex vivo cardiac performance but also exerts antioxidative, anti-inflammatory, and anti-apoptotic effects, accompanied by significant modulation of the JAK2/STAT3 signaling pathway. Overall, these data indicate that the superior functional benefits of dual therapy arise from multilayered cellular protection rather than isolated pathway modulation and underscore the translational relevance of dual SGLT2i–ARNI treatment in modern HF management. Further studies investigating protein-level signaling, mitochondrial function, and long-term remodeling are warranted to fully delineate the therapeutic potential and mechanistic interactions of these agents.

## Figures and Tables

**Table 1:** RT-PCR primers utilized in this study.

Target Gene	Left Primer (Forward)	Right Primer (Reverse)
β-actin	GATCAGCAAGCAGGAGTACGAT	GTAACAGTCCGCCTAGAAGCAT
eNOS	GAGGGAGTCAGCCTAAATCCTG	ATCAAAGCATACGAAGAGGGCA
iNOS	TCAGGCTTGGGTCTTGTTAAGC	CTTGTGGTGAAGGGTGTCGT
TNF-α	GAAAGCATGATCCGAGATGTGG	CAGGAATGAGAAGAGGCTGAGG
IL-6	GATACCACCCACAACAGACCAG	GTGCATCATCGCTGTTCATACA
IL-1β	GCAAGTGTCTGAAGCAAGCTATG	TCTGTCAGCCTCAAAGAACAGG
IL-17	GCAAGAGATCCTGGTCCTGAAG	AGGTCTCTGTTTAGGACGCATG
IL-10	CTTACTGGCTGGAGTGAAGACC	CTGGGAAGTGGGTGCAGTTATT
IL-13	GCAAGTGTCTGAAGCAGCTATG	TCTGTCAGCCTCAAAGAACAGG
Bcl-2	GCAAAGCACATCCAATAAAAGCG	GTACTTCATCACGATCTCCCGG
Bax	GCTACAGGGTTTCATCCAGGAT	ATGTTGTTGTCCAGTTCATCGC
CASP-3	GGAAGATCACAGCAAAAGGAGC	GCAGTAGTCGCCTCTGAAGAAA
CASP-9	TGTACTCCAGGGAAGATCGAGA	CGTTGTTGATGATGAGGCAGTG
JAK2	TCCACCCAATCATGTCTTCCA	ATGGTGTGCATCCGCAGTTA
STAT3	CTGAGGTACAATCCCGCTCG	TCGGTCAGTGTCTTCTGCAC
mTOR	TGCTGGTGTCCTTTGTGAAG	TTGTGCTCTGGATTGAGGTG
PPARγ	TTCAGAAGTGCCTTGCTGTG	CCAACAGCTTCTCCTTCTCG

Abbreviations: RT-PCR, real-time quantitative polymerase chain reaction; eNOS, endothelial nitric oxide synthase; iNOS, inducible nitric oxide synthase; TNF-α, tumor necrosis factor α; IL-6, interleukin-6; IL-1β, interleukin-1β; IL-17; interleukin-17; IL-10, interleukin-10; IL-13, interleukin-13; Bcl-2, B-cell lymphoma 2; Bax, Bcl-2-associated X protein; CASP-3, Caspase-3; CASP-9, Caspase-9; JAK2, Janus kinase 2; STAT3, signal transducer and activator of transcription; mTOR, mammalian target of rapamycin; PPARγ, Peroxisome proliferator-activated receptor γ.

## Data Availability

The original contributions presented in this study are included in the article/Supplementary Materials. Further inquiries can be directed to the corresponding author.

## Supplementary Materials

The following supporting information can be downloaded at: https://www.mdpi.com/article/10.3390/biomedicines14051115/s1, Figure S1: Effect of the applied therapeutic protocols (within-group) on the value of the maximum rate of pressure development in the left ventricle (dp/dt max) of the hearts of rats with heart failure. Figure S2: Effect of the applied therapeutic protocols (within-group) on the value of the minimum rate of pressure development in the left ventricle (dp/dt min) of the hearts of rats with heart failure. Figure S3: Effect of the applied therapeutic protocols (within-group) on the value of systolic pressure in the left ventricle (SLVP) of the hearts of rats with heart failure. Figure S4: Effect of the applied therapeutic protocols (within-group) on the value of diastolic pressure in the left ventricle (DLVP) of the hearts of rats with heart failure. Figure S5: Effect of the applied therapeutic protocols (within-group) on heart rate (HR) in rats with heart failure. Figure S6: Effect of the applied therapeutic protocols (within-group) on coronary flow (CF) in rats with heart failure. Figure S7: Effect of the applied therapeutic protocols (within-group) on the lipid peroxidation index (measured as TBARS) in the coronary venous effluent of rats with heart failure during two consecutive autoregulation protocols. Figure S8: Effect of the applied therapeutic protocols (within-group) on nitrite (NO_2_^−^) concentration in the coronary venous effluent of rats with heart failure during two consecutive autoregulation protocols. Figure S9: Effect of the applied therapeutic protocols (within-group) on superoxide anion radical (O_2_^−^) concentration in the coronary venous effluent of rats with heart failure during two consecutive autoregulation protocols. Figure S10: Effect of the applied therapeutic protocols (within-group) on hydrogen peroxide (H_2_O_2_) concentration in the coronary venous effluent of rats with heart failure during two consecutive autoregulation protocols.

## Abbreviations

The following abbreviations are used in this manuscript:

ANP	Atrial natriuretic peptide
ARNI	Angiotensin receptor–neprilysin inhibitor
Bax	Bcl-2-associated X protein
Bcl-2	B-cell lymphoma 2
BNP	B-type natriuretic peptide
BW	Body weight
cDNA	Complementary DNA
CF	Coronary flow
CK-MB	Creatine kinase MB isoform
CPP	Coronary perfusion pressure
CTRL	Control group
CVE	Coronary venous effluent
DLVP	Diastolic left ventricular pressure
dp/dt max	Maximum rate of left ventricular pressure development
dp/dt min	Minimum rate of left ventricular pressure development
EF	Ejection fraction
ELISA	Enzyme-linked immunosorbent assay
eHF	Experimental heart failure
eNOS	Endothelial nitric oxide synthase
HF	Heart Failure
HF-EMPA	Heart failure group treated with empagliflozin
HF- S/V	Heart failure group treated with sacubitril/valsartan
HF-EMPA/S/V	Heart failure group treated with empagliflozin and sacubitril/valsartan
HFrEF	Heart failure with reduced ejection fraction
HR	Heart rate
H_2_O_2_	Hydrogen peroxide
IL-1β	Interleukin-1 beta
IL-6	Interleukin-16
IL-10	Interleukin-10
IL-13	Interleukin-13
IL-17	Interleukin-17
iNOS	Inducible nitric oxide synthase
IRI	Ischemia–reperfusion injury
ISO	Isoproterenol
JAK2	Janus kinase 2
LV	Left ventricle/left ventricular
mTOR	Mammalian target of rapamycin
NO_2_^−^	Nitrite
O_2_^−^	Superoxide anion radical
OS	Oxidative stress
PCR	Polymerase chain reaction
PPARγ	Peroxisome proliferator-activated receptor gamma
PSR	Picrosirius Red
ROS	Reactive oxygen species
RT-PCR	Real-time polymerase chain reaction
SD	Standard deviation
SERCA2a	Sarcoplasmic reticulum Ca^2+^-ATPase 2a
SLVP	Systolic left ventricular pressure
SGLT2	Sodium-glucose co-transporter 2
STAT3	Signal transducer and activator of transcription 3
TBARS	Thiobarbituric acid reactive substances
TNF-α	Tumor necrosis factor alpha
TTE	Transthoracic echocardiography
