# Supplementary figures and images for: Dual Empagliflozin and Sacubitril/Valsartan Therapy Improves Ex Vivo Cardiac Function in a Rat Model of Heart Failure
Source: Biomedicines. 2026 May 14;14(5):1115. doi: 10.3390/biomedicines14051115 (PMC13204210; doi:10.3390/biomedicines14051115)

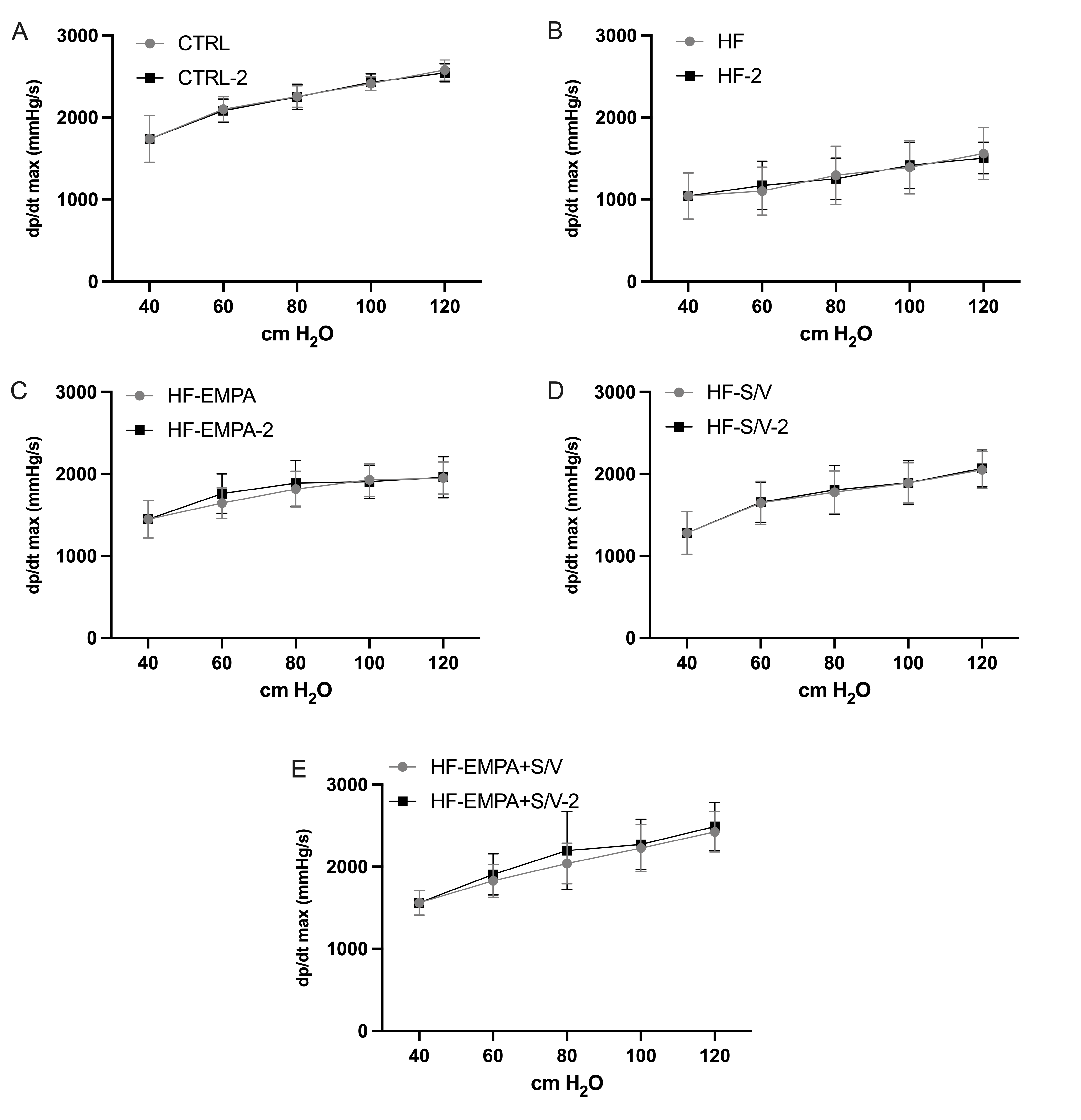

Supplement: Supplementary file 1 [file biomedicines-14-01115-s001.zip › Supplementary figures/Figure S1.tiff]

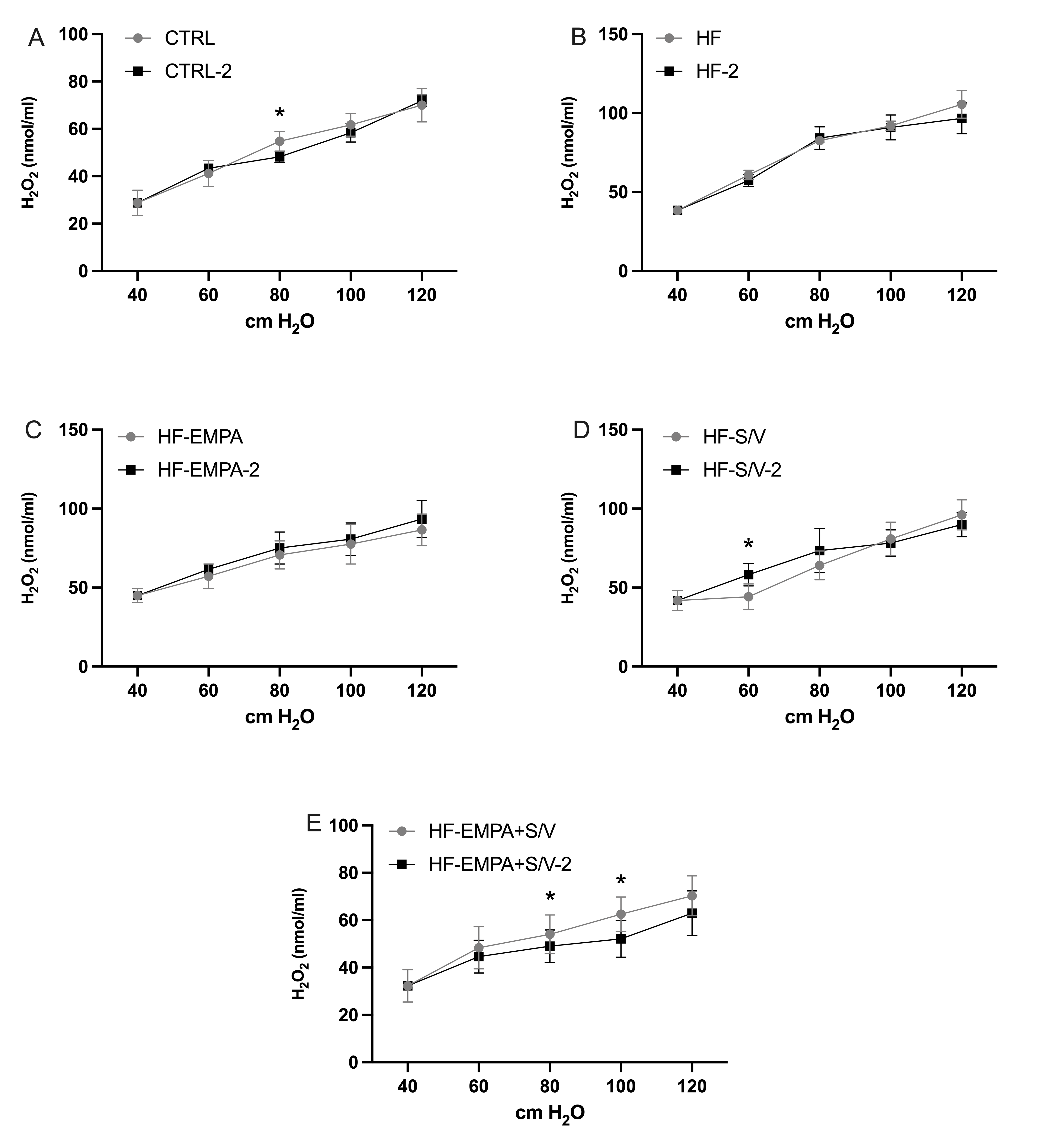

Supplement: Supplementary file 1 [file biomedicines-14-01115-s001.zip › Supplementary figures/Figure S10.tiff]

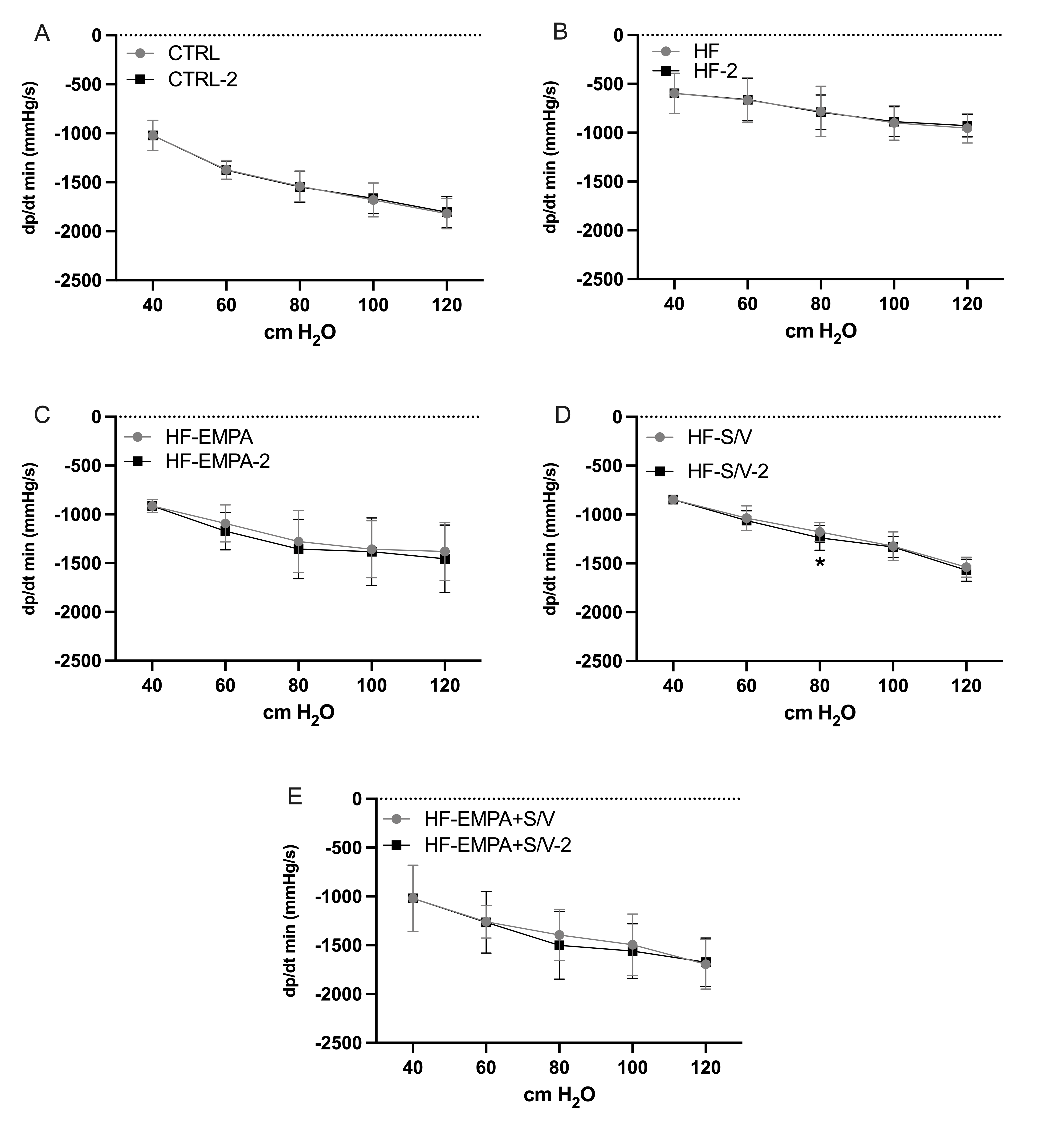

Supplement: Supplementary file 1 [file biomedicines-14-01115-s001.zip › Supplementary figures/Figure S2.tiff]

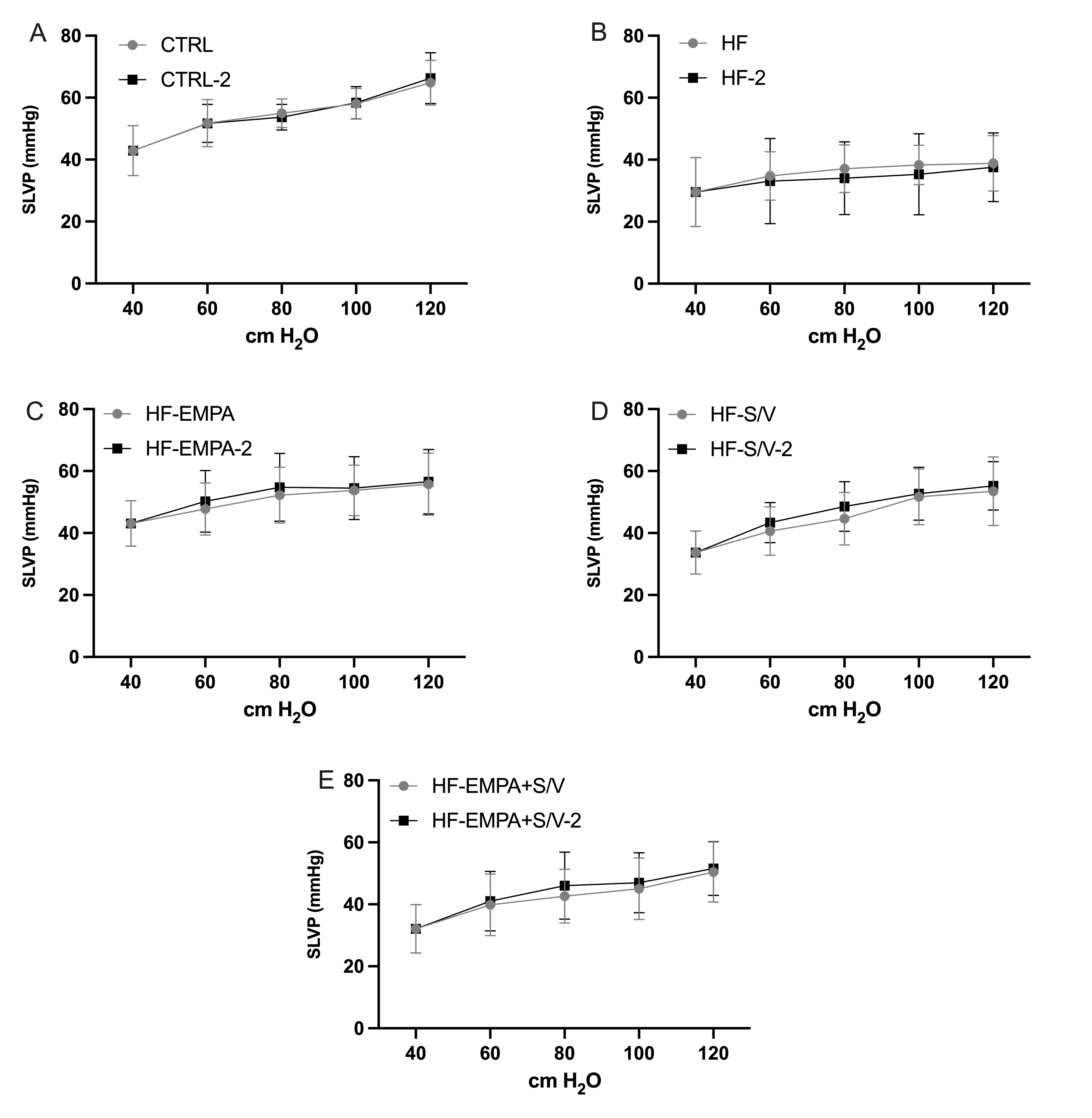

Supplement: Supplementary file 1 [file biomedicines-14-01115-s001.zip › Supplementary figures/Figure S3.tiff]

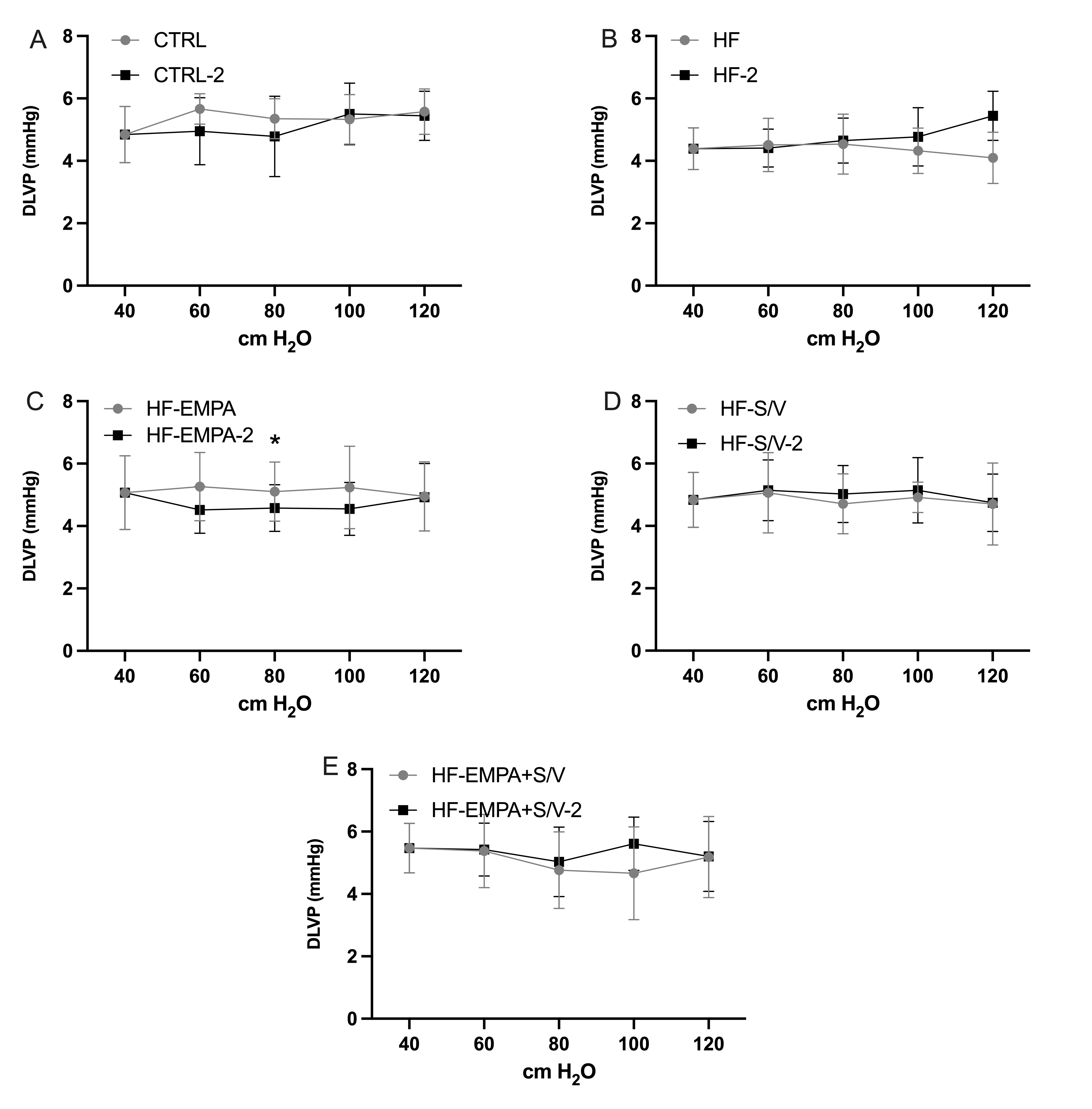

Supplement: Supplementary file 1 [file biomedicines-14-01115-s001.zip › Supplementary figures/Figure S4.tiff]

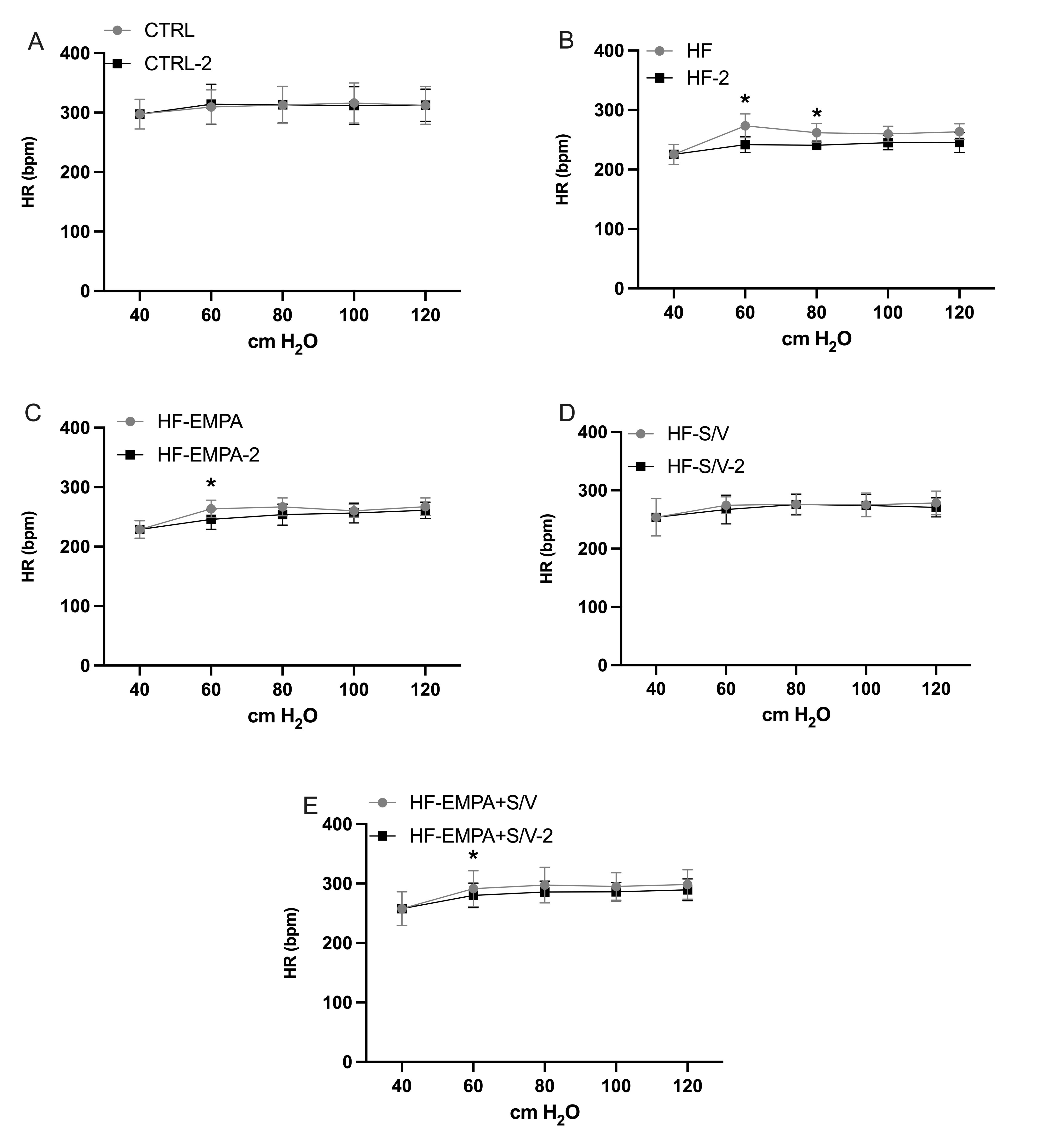

Supplement: Supplementary file 1 [file biomedicines-14-01115-s001.zip › Supplementary figures/Figure S5.tiff]

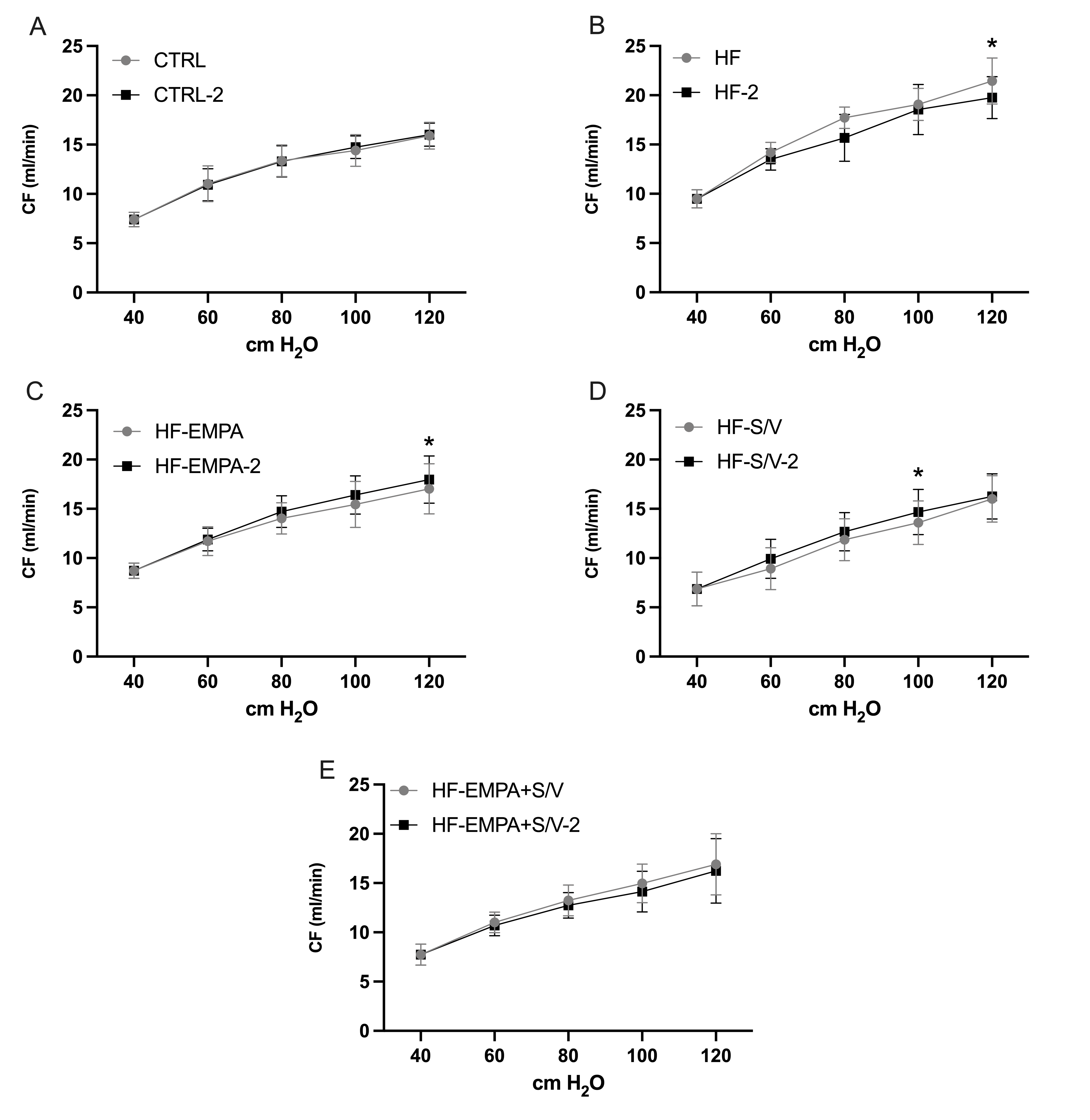

Supplement: Supplementary file 1 [file biomedicines-14-01115-s001.zip › Supplementary figures/Figure S6.tiff]

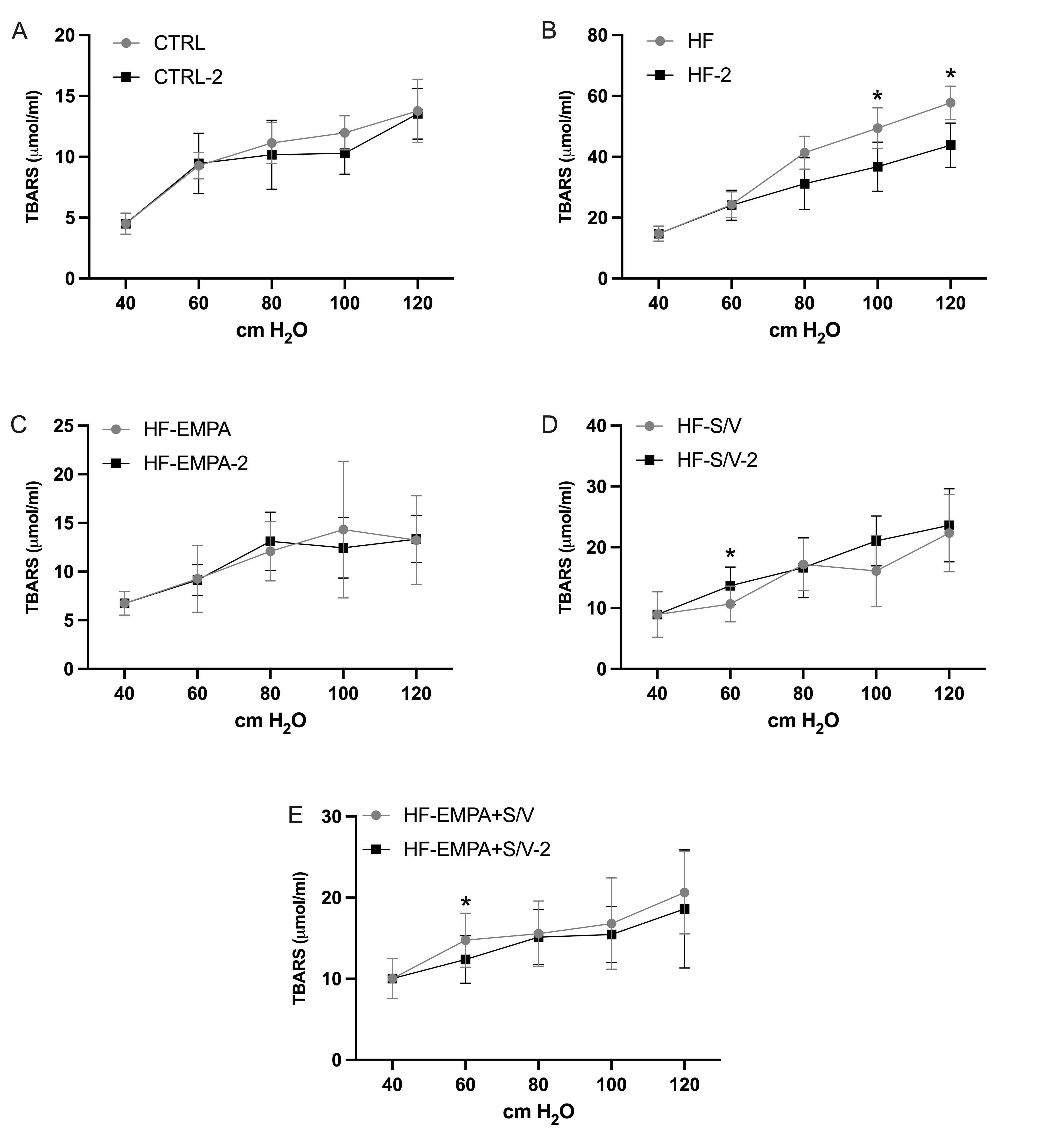

Supplement: Supplementary file 1 [file biomedicines-14-01115-s001.zip › Supplementary figures/Figure S7.tiff]

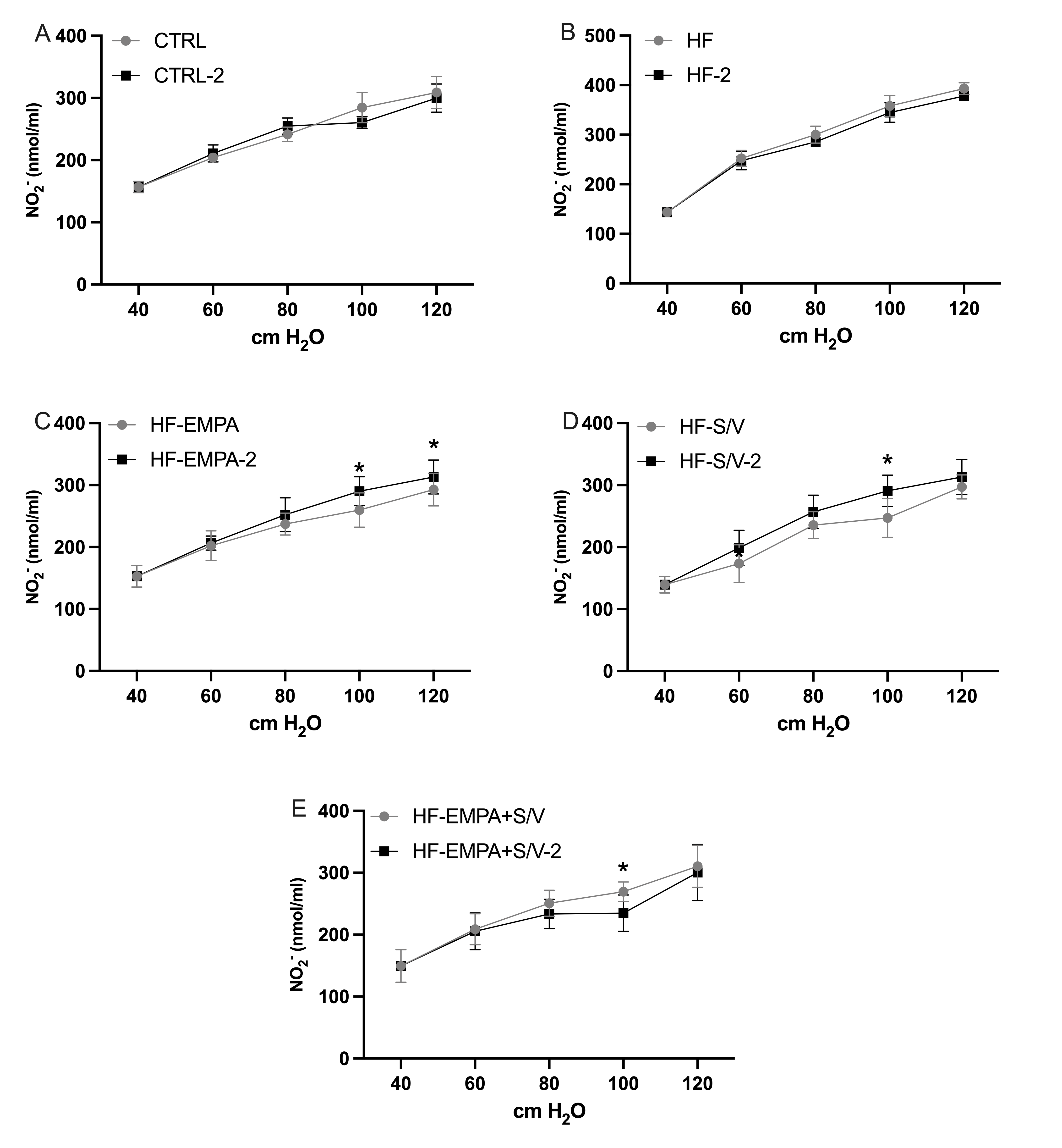

Supplement: Supplementary file 1 [file biomedicines-14-01115-s001.zip › Supplementary figures/Figure S8.tiff]

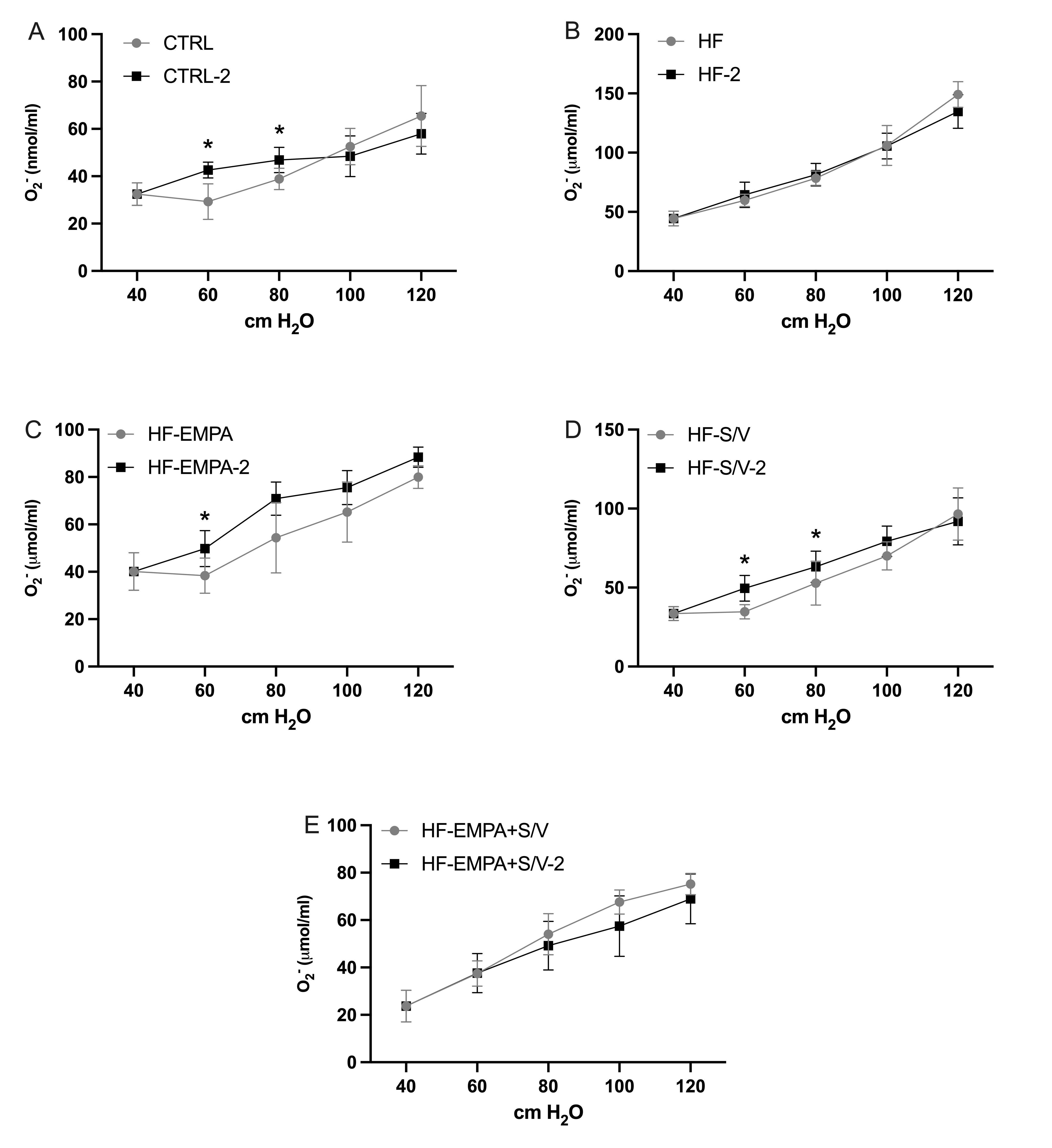

Supplement: Supplementary file 1 [file biomedicines-14-01115-s001.zip › Supplementary figures/Figure S9.tiff]
